# Comparative Transcriptomic Analysis of *Streptococcus thermophilus* TH1436 and TH1477 Showing Different Capability in the Use of Galactose

**DOI:** 10.3389/fmicb.2018.01765

**Published:** 2018-08-07

**Authors:** Sabrina Giaretta, Laura Treu, Veronica Vendramin, Vinícius da Silva Duarte, Armin Tarrah, Stefano Campanaro, Viviana Corich, Alessio Giacomini

**Affiliations:** ^1^Department of Agronomy Food Natural Resources Animal and Environment, University of Padova, Padova, Italy; ^2^Department of Environmental Engineering, Technical University of Denmark, Kongens Lyngby, Denmark; ^3^Department of Microbiology, Universidade Federal de Viçosa, Viçosa, Brazil; ^4^Department of Biology, University of Padova, Padova, Italy

**Keywords:** Galactose metabolism, RNA-seq, comparative transcriptome analysis, CcpA, *gal-lac* operon, mixed acid fermentation

## Abstract

*Streptococcus thermophilus* is a species widely used in the dairy industry for its capability to rapidly ferment lactose and lower the pH. The capability to use galactose produced from lactose hydrolysis is strain dependent and most of commercial *S. thermophilus* strains are galactose-negative (Gal^−^), although galactose-positive (Gal^+^) would be more technologically advantageous because this feature could provide additional metabolic products and prevent galactose accumulation in foods. In this study, a next generation sequencing transcriptome approach was used to compare for the first time a Gal^+^ and a Gal^−^ strain to characterize their whole metabolism and shed light on their different properties, metabolic performance and gene regulation. Transcriptome analysis revealed that all genes of the *gal* operon were expressed very differently in Gal^+^ and in the Gal^−^ strains. The expression of several genes involved in mixed acid fermentation, PTS sugars transporter and stress response were found enhanced in Gal^+^. Conversely, genes related to amino acids, proteins metabolism and CRISPR associated proteins were under-expressed. In addition, the strains showed a diverse series of predicted genes controlled by the transcriptional factor catabolite control protein A (CcpA). Overall, transcriptomic analysis suggests that the Gal^+^ strain underwent a metabolic remodeling to cope with the changed environmental conditions.

## Introduction

Lactose is the main carbon and energy source for *Streptococcus thermophilus* and it is metabolized by fermentation into lactic acid. This process represents a very important step in most dairy transformations, since the vast majority of cheeses contain this bacterial species, with limited exceptions (Pogačić et al., 2010). *S. thermophilus* metabolism rapidly lower the pH and, since it is known that pH decrease induces modification in the bacterial population composition (Bovo et al., 2012; Maragkoudakis et al., 2013) this becomes a key factor for the quality and safety of cheeses and dairy products (Leroy and De Vuyst, 2004). Lactose is imported into the cell by a lactose permease (LacS) and is further hydrolyzed to glucose and galactose by a β-galactosidase (LacZ) (Schroeder et al., 1991; Foucaud and Poolman, 1992; Gunnewijk et al., 2001). While glucose is used by the glycolytic pathway, galactose is not metabolized in most known *S. thermophilus* strains and it is secreted in the medium by the LacS antiporter, thus allowing the uptake of additional lactose from the medium (Gunnewijk et al., 2001; Cochu et al., 2005). Galactose accumulation in dairy products can lead to several unfavorable events, e.g., browning on heat-treated products such as Mozzarella in pizza preparation, cheese fractures due to CO_2_ overproduction by heterofermentative bacteria and toxic effects on people affected by galactosemia, a genetic disease affecting galactose metabolism (Wu et al., 2015). The availability of starter strains capable of utilizing galactose could be therefore beneficial and of extreme interest for dairy productions (Rao et al., 2004; Anbukkarasi et al., 2014) and also for fermentation of their byproducts (Levander and Rådström, 2001; Price et al., 2012). In galactose positive (Gal^+^) strains, this sugar is metabolized by the Leloir pathway, that includes four enzymes, namely galactose mutarotase (GalM), galactokinase (GalK), galactose-1-phosphate uridylyltransferase (GalT) and UDP-glucose 4-epimerase (GalE) (Levander and Svensson, 2002). In *S. thermophilus* the five genes related to galactose metabolism (*galR, galK, galT, galE, galM*) are located upstream of the *lac* operon (*lacSZ*). Genes *galK, galT, and galE* are under the control of the same promoter and constitute the *gal* operon (Vaughan et al., 2001). The single nucleotide polymorphisms (SNPs) localized in the *galKTE* promoter was proposed as the cause of the low efficiency in initiating the *galKTE* operon transcription leading to the inability to metabolize galactose for most *S. thermophilus* strains (galactose-negative strains; Gal^−^) (Vaughan et al., 2001).

Nowadays, a transcriptomic comparison to identify differences between Gal^+^ and Gal^−^ strains is still lacking. Moreover, although both physiological features and genetic bases of *S. thermophilus* metabolism have been well studied, there is still little information available on the expression of genes involved in technologically-relevant metabolic pathways that could influence strains performance during the manufacturing processes. The present study compares for the first time the transcriptomic profiles of a Gal^+^ and a Gal^−^ *S. thermophilus* strains using the most advanced next generation sequencing (NGS) techniques for genes expression analysis (RNA sequencing, RNA-seq). Transcriptome analysis highlighted differences not only in gene expression of lactose and galactose related genes, but also shed light on regulatory effects on genes involved in other energetic metabolisms and biological processes, such as mixed acid fermentation and stress response. Expression changes in the Gal^+^ strain were also associated with the putative involvement of CcpA, the most important pleiotropic transcription factor in carbon catabolite control (Deutscher, 2008), suggesting a more complex scenario.

## Materials and methods

### Strains and growth conditions

Six *S. thermophilus* strains isolated in Italy with publicly available genomes (Treu et al., 2014b,c) were selected for the present study (Table 1). Strains were routinely grown overnight at 37°C in M17 broth (Oxoid, UK) supplemented with 0.5% (w/v) lactose (M17lac), unless otherwise stated.

**Table 1:** *S. thermophilus* strains origin and optical density (OD_600_) values after 24 h of growth in M17 containing 1% galactose.

**Strain**	**OD_600_ values**	**Geographical region**	**Isolation matrix**	**Animal**	**References**
TH1436	0.67 ± 0.08	Friuli Venezia Giulia	Raw milk	Goat	Treu et al., 2014c
TH1435	0.28 ± 0.02	Friuli Venezia Giulia	Raw milk	Goat	Treu et al., 2014c
TH1477	0.21 ± 0.03	Veneto	Raw milk	Cow	Treu et al., 2014b
1F8CT	0.09 ± 0.01	Veneto	Curd from raw milk	Cow	Treu et al., 2014b
TH982	0.24 ± 0.09	Campania	Mozzarella curd	Buffalo	Treu et al., 2014b
TH985	0.28 ± 0.07	Campania	Mozzarella whey	Buffalo	Treu et al., 2014b

*Values are means ± standard deviations from 3 independent experiments with 4 technical replicates each*.

### Growth on galactose and sugar utilization

The ability to grow on galactose-containing medium was monitored using a microtiter plate reader (Tecan, Austria GmbH, Grödig), by recording the optical density (OD) at 600 nm. The experiment was repeated 3 times with 4 technical replicates each. Negative controls were also added to the experiment. A loopful of frozen stock culture was transferred into a 15-mL sterile tube containing 10 mL of M17lac and incubated at 37°C for 24 h. Cells were then collected by centrifugation for 10 min at 10,000 rpm and the pellet washed twice with 2 mL of PBS, resuspended in fresh M17 broth supplemented with 1% galactose to a final concentration of 10^6^ cells/mL. Aliquots of 200 μl were then transferred into 96-well microtiter plate wells (Sigma SIAL0596, MO, USA) and incubated at 37°C for 24 h inside the plate reader.

Lactose and galactose consumption were measured for *S. thermophilus* strains TH1436 and TH1477 grown at 37°C in tubes containing 10 mL of M17lac broth, starting from an inoculum of 10^6^ cells/mL. Aliquots of 1 mL were collected at 0, 6, 18 and 24 h and their optical density (OD_600_) was measured. Samples were centrifuged at 55,000 rpm for10 min to remove the cells and the supernatants were stored at −20°C. The concentration of lactose and galactose was determined spectrophotometrically using the Lactose/Galactose Assay Kit (Megazyme, Wicklow, Ireland) according to the manufacturer's protocol.

The acidification curves for strains TH1436 and TH1477 were performed in M17lac batch cultures at 37°C by measuring the pH value every 10 min with an immersed electrode connected to a pHmeter.

### Gal operon sequence inspection and regulatory site prediction

The structure of the *gal-lac* operon in the genome of the six strains was identified by multiple genome alignment with Progressive Mauve software (Darling et al., 2010), using the *gal-lac* operon sequence of strain LMG13811 (Bolotin et al., 2004) as reference. The relative positions of genes and regulatory elements in the operon were manually compared among strains. Subsequently, nucleotide and amino acid sequences for each gene were aligned with Clustal Omega (Sievers et al., 2011) and visualized with the alignment editor Bioedit (Hall, 1999). Multi-alignment of the intergenic regions was manually inspected to identify nucleotide polymorphisms among strains.

The *cre* site prediction was performed using the *S. thermophilus* CNRZ302 strain weight matrix (HWNMHAHSVNDHNHHN; consensus sequence ATGAAAACGTTTTCAA), predicted and obtained from RegPrecise (http://regprecise.lbl.gov) (Novichkov et al., 2013). The weight matrix motif was used to screen the whole genome of TH1436 and TH1477 using the FIMO tool of MEME Suite software (http://meme-suite.org) (Bailey et al., 2009). The resulting list of potential *cre* sites was refined by selecting only predictions having a *p*-value lower than 10^−6^. Subsequently, genes putatively regulated by the predicted *cre* sites were manually identified by Artemis browser (Carver et al., 2012).

### RNA extraction and sequencing

For gene expression analysis, strains were grown for 18 h, to full stationary phase, at 37°C in 50 mL of M17lac, starting from a 10^6^ cells/mL inoculum. A minimum of 10^10^ cells were harvested by centrifugation at 5,000 rpm for 5 min at 4°C in three replicates, pellets were snap frozen using liquid nitrogen and stored at −80°C until RNA extraction. Frozen cells were lysed by adding 2 mL of lysozyme solution (10 mM Tris-HCl, 0.1 mM EDTA, 15 mg/mL lysozyme, pH 8.0) and vortexed until resuspension for about 30 s. Ten μl of 10% (w/v) SDS were then added to the sample and incubated for 5 min at room temperature. Afterwards, 350 μl of freshly prepared 1% (v/v) 2-mercaptoethanol lysis buffer and 3 mL of TRIzol reagent (Invitrogen, Rodano, IT) were added and the sample was vortexed for 15 min with 50 mg of 0.6 mm cold glass beads (Sigma, Missouri, USA). Chloroform (600 μl) was added and the mixture was centrifuged at 12,000 rpm for 20 min at 4°C. The upper phase was collected and RNA was extracted using the Purelink RNA minikit following the manufacturer's protocol, including a DNAse PureLink (Invitrogen, Rodano, IT) treatment. RNA quantification was performed using both NanoDrop (ThermoFisher Scientific, Waltham, MA, USA) and Qubit (ThermoFisher Scientific). RNA integrity was checked both on denaturing agarose gel and by the Bioanalyzer RNA 6000 Pico chip (Agilent Technologies). Finally, ribosomal RNA was depleted using the MICROBExpress kit (Ambion, Rodano, IT) following manufacturer's instructions.

Samples were sequenced at the Ramaciotti Centre for Gene Function Analysis (University of New South Wales, Sydney, AU) using the Illumina Nextseq 500 platform (Illumina, San Diego, CA, USA), generating 75 bp paired-end reads. Libraries with insert size between ~350 bp and 1.5 Kbp were produced using the TruSeq RNA Library Preparation kit (Illumina, San Diego, CA, USA).

### Transcriptomic profiles reconstruction and gene expression evaluation

Raw data quality check and filtering were performed using the CLC Main Workbench 7.6.4 (CLC bio, Waltham, MA, USA) with quality score higher than 0.05 and reads lengths greater than 73 bp. Only reads with one best-hit place were kept for the analysis. Total mapped reads per gene were calculated in order to normalize and compare expression levels within a sample or between different samples (reads per kilobase per million mapped reads, RPKM). Since *S. thermophilus* genomes were annotated using SEED subsystems database, orthologous genes between strains were identified using the RAST genome comparison tool (Aziz et al., 2008). Differential gene expression between strains was determined by calculating the fold change, applying the tagwise dispersion with CLC Main Workbench 4.7.6 (CLC bio, Waltham, MA, USA) and *p*-values were calculated using edgeR software (Zhou et al., 2014). The significance threshold used was “*p*-value < 0.05.” Genes were considered differentially expressed when the fold change was 2 or higher. Most differentially expressed (DE) genes were assigned to functional categories according to the SEED annotation. The 64 DE unclassified genes were manually assigned to the “first level” SEED categories after functional prediction based on BLASTp results in UniProt (Apweiler, 2004) (http://www.uniprot.org/blast/). However, 17 of them did not show any similarity with known proteins and were therefore assigned to the group “hypothetical proteins.”

Transcript reconstruction and operon prediction were performed using approaches previously described (Sardu et al., 2014; Taha et al., 2016). Briefly, the log_2_ ratio of the coverage for each couple of neighboring genes was calculated and, if lower than one standard deviation (determined for the entire distribution of the log_2_ ratios), they were considered part of the same operon. Operon reconstruction was obtained excluding genes separated by regions of zero coverage in the intergenic regions and genes with very low coverage (lower than 2). Coverage profiles at single base level were determined using the ORA software (Sardu et al., 2014). Regions upstream and downstream of each predicted operon were inspected in order to locate positions of very rapid coverage reduction, indicating transcription start/end sites. Details regarding the applied procedure and perl scripts used for the analysis can be found in sourceforge (https://sourceforge.net/projects/trb/).

Nucleotide sequence accession number. The RNA-seq data have been deposited in SRA DDBJ/ENA/GenBank under the Biproject with accession no PRJNA412475 (Biosamples SAMN07714826 and SAMN07714827). The raw sequence data are available at Sequence Read Archive (SRA of NCBI) with accession no SRR6112518, SRR6112538, SRR6112863 (for SAMN07714826) and SRR6112864, SRR6112865, SRR6112866 (for SAMN07714827).

### Statistical analysis

The SigmaPlot software version 12.0 (Systat Software, San Jose, CA) was used for statistical analysis of sugar consumption. After normality test, data were analyzed for statistical significance using analysis of variance (ANOVA) followed by Tukey's test.

To identify the SEED functional categories statistically enriched of DE genes, 10,000 random gene samplings were performed using all the protein encoding genes as dataset. Resampling was performed with a custom perl script implementing the “rand()” function as previously described (Treu et al., 2014a). Briefly, for each category the fraction of random samples in which the number of randomly sampled genes (RS) was equal to or higher than the DE was calculated (RS =< DE). If this fraction was lower than the significance level α (0.05) the enrichment of the specific functional category was considered significant.

## Results and discussion

### Growth kinetics and sugar consumption

The growth of *S. thermophilus* strains in M17 medium with galactose as energy source was evaluated (Table 1). After 24 h at 37°C only strain TH1436 grew well and reached an OD_600_ of 0.67 while the remaining strains displayed a faint turbidity, up to OD_600_ of 0.28, which is equivalent to that obtained in M17 without any sugar added (data not shown) that we therefore consider as negative. Since in Gal^+^ strains galactose is metabolized after lactose hydrolysis while in Gal^−^ strains it is exported outside the cell (Poolman, 1993; Vaillancourt et al., 2002), the capability to use galactose was determined by quantifying the galactose released in the growth medium (Table 2). In parallel, lactose in the medium was also quantified, to follow its consumption. Two strains were tested, namely *S. thermophilus* TH1436 (Gal^+^), the only one able to use galactose as energy source, and *S. thermophilus* TH1477 (Gal^−^), chosen as a representative of strains not utilizing galactose. After 6 h (t6), 48% of the initial lactose was still present in the medium of the Gal^+^ strain, whereas the Gal^−^ already utilized 48% of it. Concurrently, the level of galactose in the medium increased for both strains, indicating that also the Gal^+^ exported galactose during the first 6 h. After 18 h (t18) both strains consumed all the lactose and galactose was no longer present in the Gal^+^ medium, while its level doubled in the Gal^−^ culture. This is in agreement with other reported (Vin et al., 2005) behaviors of Gal^−^ phenotypes during growth on lactose, because galactose is exported by the LacS lactose-galactose antiporter system and accumulates in the medium. From 18 h onwards the level of galactose remained unchanged. However, the amount of galactose measured was less than stoichiometrically expected from the internalized lactose (i.e., 1:2), in accordance with previous findings (Vaillancourt et al., 2002; Vin et al., 2005). Apart from the fact that the Gal^−^ strains maintains a weak capability to use galactose, this discrepancy could be attributed to the up-regulation of the enzymes of the Leloir pathway by lactose starvation, as it was observed in the Gal^−^ *S. thermophilus* proteome (Arena et al., 2006). However, the mechanism of induction has not been characterized yet. During the period from 6 to 18 h the Gal^+^ strain internalized the previously exported galactose, but this energy surplus does not appear to have been used for increasing cells number. Finally, sugar amounts did not change significantly after 18 h for both strains, indicating a steady metabolic activity during that period, corresponding to the stationary phase of growth.

**Table 2:** Optical density (OD_600_) of TH1436 (Gal^+^) and TH1477 (Gal^−^) cultures grown in M17lac and amounts of lactose and galactose in the medium.

	**Strain**	**Time (h)**			
		**t0**	**t6**	**t18**	**t24**
OD_600_	TH1477	0.01 ± 0.00	1.09 ± 0.09	1.97 ± 0.02	1.98 ± 0.02
	TH1436	0.01 ± 0.00	0.80 ± 0.01	1.90 ± 0.01	1.91 ± 0.01
Lactose (g/L)	TH1477	5.12 ± 0.03	1.07 ± 0.22***	0.01 ± 0.01	0.04 ± 0.04
	TH1436	4.83 ± 0.16	2.30 ± 0.14	0.02 ± 0.01	0.04 ± 0.02
Galactose (g/L)	TH1477	0.02 ± 0.01	0.18 ± 0.02*	0.37 ± 0.04***	0.34 ± 0.04***
	TH1436	0.04 ± 0.02	0.15 ± 0.00	0.06 ± 0.03	0.04 ± 0.02

*Values are means ± standard deviations obtained from 3 independent experiments. Asterisks indicate statistically significant differences between strains (ANOVA, Tukey's test)*.

**Figure 1:**
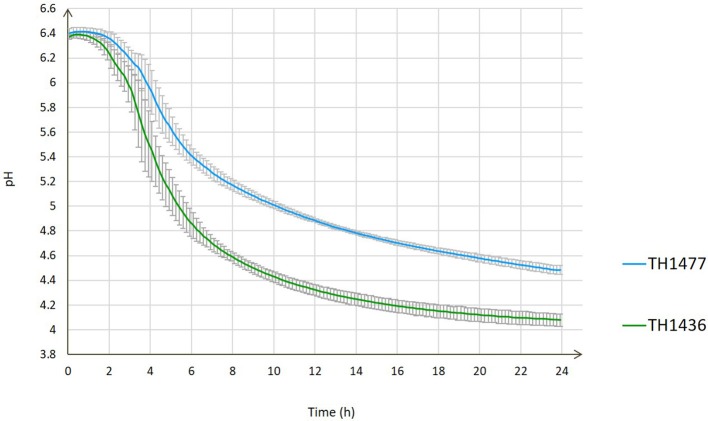
Evolution of pH value during 24 h of growth (acidification curves) of *S. thermophilus* strains TH1436 and TH1477.

During their growth, strains release in the medium acids, produced by fermentation, thus determining a decrease in the pH value. Since acidic environments constitute a stressful condition for bacterial metabolism, we performed the acidification curves for the two strains (Figure 1). It can be noted that after 18 h (the sampling point for the RNA analysis) the strains had almost completed their acidification activity. The Gal^+^ strain showed a faster acidification rate and capability since it reached pH 4.18 while the Gal^−^ stopped at 4.62, thus creating a less stressful environment for the Gal^−^ strain. Since it is known that acidification activity can be positively influenced by the presence of the cell envelope proteinase (PrtS) in *S. thermophilus* (Dandoy et al., 2011), it must be ascertained that strains TH1436 and TH1477 are in the same condition regarding the presence/absence of this gene in their genome. In a previous study (Vendramin et al., 2017), we evidenced that only TH1435, among the strains used in this work, contains a complete *prtS* sequence (identity = 97%, gaps = 5/4582, *E*-value = 0.0). Although short sequences are present in all strains, their coverage (< 20%) was considered insufficient for gene functionality. Finally, a phenotypic assay performed on plate (Morris et al., 2012) showed absence of proteinase activity in all strains (data not shown), thus excluding a PrtS involvement in the diverse acidification capabilities recorded between TH1436 and TH1477.

### *gal-lac* operon structure

**Figure 2:**
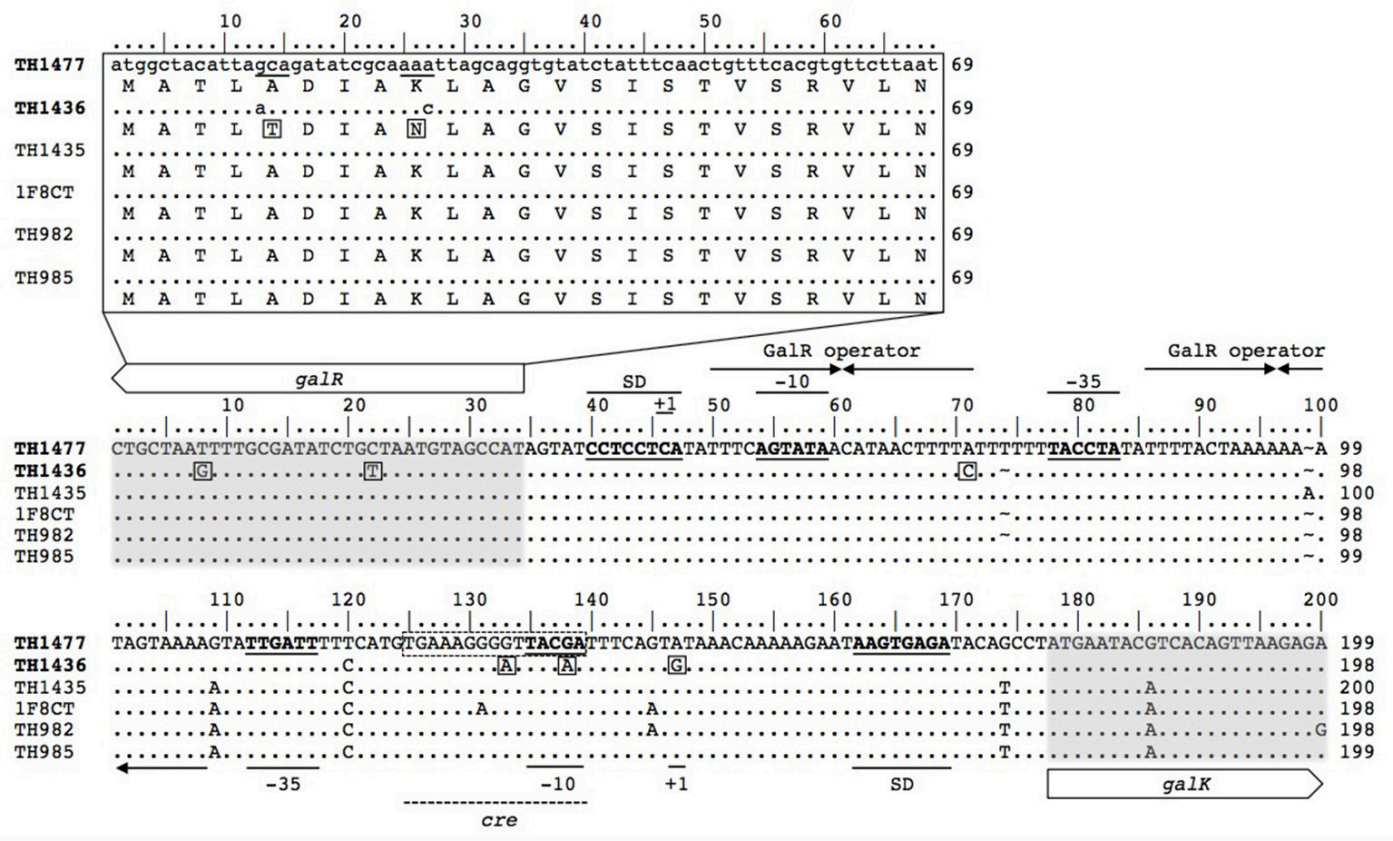
Nucleotide sequences alignment of the *galR*-*galK* intergenic region and amino acid sequence alignment of the *galR* DNA binding site. Alignment of the *galR*-*galK* intergenic sequences of the 6 strains. Strain TH1477 (Gal^−^) was used as reference sequence followed by strain TH1436 (Gal^+^). The beginning of both *galR* and *galK* genes are highlighted in light gray. Promoter sequences were identified according to Vaughan et al. (2001) and Vaillancourt et al. (2002). The −35 and −10 sequences and the ribosome binding site (SD) are in bold and underlined. Transcriptional start sites are defined as +1 and the arrows indicate the inverted repeated sequences of the *GalR* operator. The conserved nucleotides compared to the sequence of strain TH1477 are represented by dots, while nucleotide substitutions are shown with letter. Variations considered important for the Gal^+^ phenotype and the *cre* site are square framed with solid and dashed line respectively. On the top the amino acid sequences alignment of the *galR* DNA binding site is presented magnified.

The alignment of the *gal-lac* operon sequence of all strains was performed (see Figure S1 online). All possess the 7 genes (*galR-KTE-M* and *lacSZ*) for galactose and lactose metabolism. Moreover, for each gene, several SNPs were present among strains, having diverse predicted effects, including missense mutations. Amino acids substitutions were more abundant in the Gal^+^ (TH1436) than in the Gal^−^ with respect to the reference strain LMG13811 (Table 3), as reported also by Vin et al. (2005), in particular regarding *galK, galT* and *galE* genes. An interesting finding was the presence of a two-amino-acids substitution in the DNA-binding site of *galR* (Figure 2). These variations can have a direct influence on the transcriptional regulation of the Gal^+^ strain, since GalR acts both as transcriptional activator on *gal-lac* operon and is also involved in a feedback loop regulation of its own expression level (Vaughan et al., 2001).

**Table 3:** Amino acids substitution in the gal genes cluster.

**Strain**	***galR***											**Sub**	***galK***														**Sub**
TH1436	**T**	**N**	V	**–**	Y	S	S	D	D	L	E	**2**	**A**	A	**L**	A	**S**	L	W	**A**	**E**	**K**	**H**	D	S	L	**7**
TH1477	A	K	V	K	Y	S	**Y**	**A**	D	**F**	E	**3**	V	A	**L**	A	**S**	L	W	**A**	**E**	**K**	Y	D	**I**	L	**6**
TH982	A	K	V	K	**C**	**I**	S	D	D	L	**G**	**3**	V	**V**	I	A	A	F	L	V	D	E	Y	D	S	L	**1**
TH985	A	K	V	K	**C**	**I**	S	D	D	L	**G**	**3**	V	A	I	A	A	F	L	V	D	E	Y	D	S	**I**	**1**
F8CT	A	K	**I**	K	Y	S	S	D	**N**	L	E	**2**	V	A	I	**T**	A	L	W	V	D	E	Y	**G**	S	L	**2**
TH1435	A	K	V	K	Y	S	S	D	D	L	E	**0**	V	A	I	**T**	A	F	L	V	D	E	Y	D	S	L	**1**
Level	:	:	:	d					:	:			.	.	:	:	:	:		.	:	:	:	.		:

**Table:** 

**Strain**	***galT***					**Sub**					***galM***					**Sub**		***galE***		**Sub**
TH1436	**G**	E	**D**	**H**	**G**	**4**	T	E	M	E	**S**	**V**	G	**G**	**I**	**4**	**R**	**V**	G	**2**
TH1477	D	**G**	G	Y	D	**1**	T	E	M	E	T	I	G	R	V	**0**	K	A	G	**0**
TH982	D	E	G	Y	D	**0**	T	E	**V**	E	T	I	S	R	V	**1**	K	A	D	**0**
TH985	D	E	G	Y	D	**0**	T	E	M	E	T	I	S	R	V	**0**	K	A	D	**0**
F8CT	D	E	G	Y	D	**0**	T	E	M	E	T	I	S	R	V	**0**	K	A	D	**0**
TH1435	D	**G**	G	Y	D	**1**	**M**	**D**	M	**D**	T	**V**	G	R	V	**3**	K	A	G	**0**
Level	.		.	:	.			:	:	:	:	:	.		:		:	.		.

*Amino acids substitutions among strains are highlighted in bold. Total numbers of substitutions (Sub) are shown for each gene of the gal operon. The bottom line indicates deletions (d) and conserved AA between groups of strongly similar properties roughly equivalent to scoring > 0.5 (:) or ≤ 0.5 and > 0 (.) in the Gonnet PAM 250 matrix*.

It was reported that the Gal^−^ phenotype is determined by an insufficient expression level of the *gal* operon genes due to the presence of variations in the *galK* promoter region (Vaughan et al., 2001; van den Bogaard et al., 2004). In this study, the comparison of *gal*-operon intergenic regions between Gal^+^ and Gal^−^ strains found differences in *galR* and *galK* (Figure 2). The promoter regions (in particular the −35 and the −10 regions) and the Shine-Dalgarno (SD) sequences were identified and manually inspected. Strain TH1436 presents a peculiar nucleotides pattern (A-A-G) at positions 133, 138, and 147, respectively, numbered as indicated in Figure 2 at the “Pribnow box” (the −10 region) of *galK* (Figure 2). Similar patterns were previously reported among Gal^+^ strains (van den Bogaard et al., 2004), in particular the “A” at position 138 was also found by Vaughan et al. (2001) and the “G” at position 147 (in correspondence of the transcription start site +1) showed a high conservation level among Gal^+^ species, since the same SNP was found in *S. salivarius* (Vaillancourt et al., 2002), a Gal^+^ species phylogenetically highly related to *S. thermophilus*. The position of the first two nucleotides of the A-A-G pattern is very interesting since it is inside a catabolite-responsive element (*cre*) that overlaps the −10 regions. In fact, *cre* is a DNA-binding sequence for CcpA which mediates the transcription of genes involved in carbon catabolism in relation to the available carbon source and cell energy demand (Deutscher, 2008). CcpA can act as repressor of the *lac* operon and activator of genes involved in glycolysis (van den Bogaard et al., 2000). However, up to now, no role has been ascribed to CcpA on *S. thermophilus gal* operon, even if some evidences in *Lactococcus lactis* suggest a positive induction of the *gal* operon transcription (Luesink et al., 1998). In this study, the *cre* sites prediction in the genome of strains TH1436 and TH1477 using the CNRZ302 strain weight matrix evidenced that the *gal* operon genes (*galK, galT, galE*) are under the control of CcpA in the Gal^+^ strain but not in the Gal^−^ (Table S3). This suggests that the only two different nucleotides found on the Gal^+^ *cre* sequence (Figure 2) are crucial for the CcpA binding site recognition, which would determine a different regulation of *gal* genes expression in this strain. However, further specific experiments on CcpA will be necessary to validate this hypothesis, which would be of great interest to clarify the regulation of the *gal* operon.

Finally, in strain TH1436 two putative operator sequences were found, based on the proposed consensus sequence of Vaughan et al. (2001) characterized by an 11-bp inverted repeated sequence with a three-nucleotide core flanked by A/T repetitions (Figure 2). Interestingly, the g.71 A > C variation found in TH1436 Gal^+^ strain might influence the *galR* repression level and could thus enhance the *galK* expression.

### Comparative transcriptional approach and transcript reconstruction

To evidence genes differentially expressed between the Gal^+^ (TH1436) and Gal^−^ (TH1477) strains, a transcriptomic analysis was performed using the RNA-seq approach. The analysis was performed at the stationary phase of the cultures, after 18 h of grow in M17lac, a condition in which the organisms need to manage efficiently the occurring metabolic changes, in particular in response to the scarce presence of energy sources. Our study was aimed at highlighting metabolic differences of two strains, one naturally endowed with the capability to metabolize galactose and one unable to do it, by comparing the expression of the whole transcriptome, not just limiting to lactose/galactose related genes. We chose to compare two natural strains, rather than creating a Gal^−^ mutant of the Gal^+^ strain, since naturally occurring Gal^−^ and Gal^+^ strains, during their evolution, could have developed differences in gene regulation in relation to their capability to metabolize galactose.

Anyway, the genetic diversity between TH1436 and TH1477 is ascribed to approximately 13,750 SNPs detected, whereas the number of total predicted genes was 1,899 and 1,986 for TH1436 and TH1477, respectively (Vendramin et al., 2017). The orthologous genes between the two strains were 1581.

Transcriptome sequencing data resulted in 1.7 × 10^7^ paired reads for each sample on average, with 1% of discarded reads after quality filtering, thus evidencing a high quality of the obtained results.

**Figure 3:**
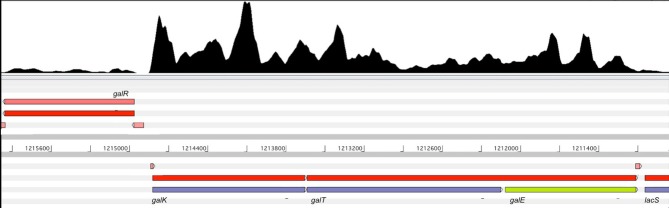
Representation of *gal* operon transcript reconstruction. On the top of the figure transcripts coverage on both strands is shown as black graph. Genes are represented by colored boxes while reconstructed transcripts are reported on a separate line represented by red boxes. The predicted UTR regions are shown as pink boxes.

Regarding the *gal* operon, transcription start sites (TSS), transcription termination sites (TTS), consensus promoter sequences and operon structures were predicted for both strains by using the RNA-seq results. Transcripts reconstruction confirmed the *gal* operon organization in *S. thermophilus*. The comparable expression levels recorded for these three transcripts confirm that they are under the control of the same promoter. However, the level of *galK* transcripts coverage was found higher than that of *galT* and *galE*, suggesting the existence of an independent transcriptional regulation of genes belonging to this operon (Figure 3). Further experiments would be of great interest to investigate whether *galK* could be regulated independently from the other constituents of the *gal* operon.

### Differentially expressed functional categories

**Figure 4:**
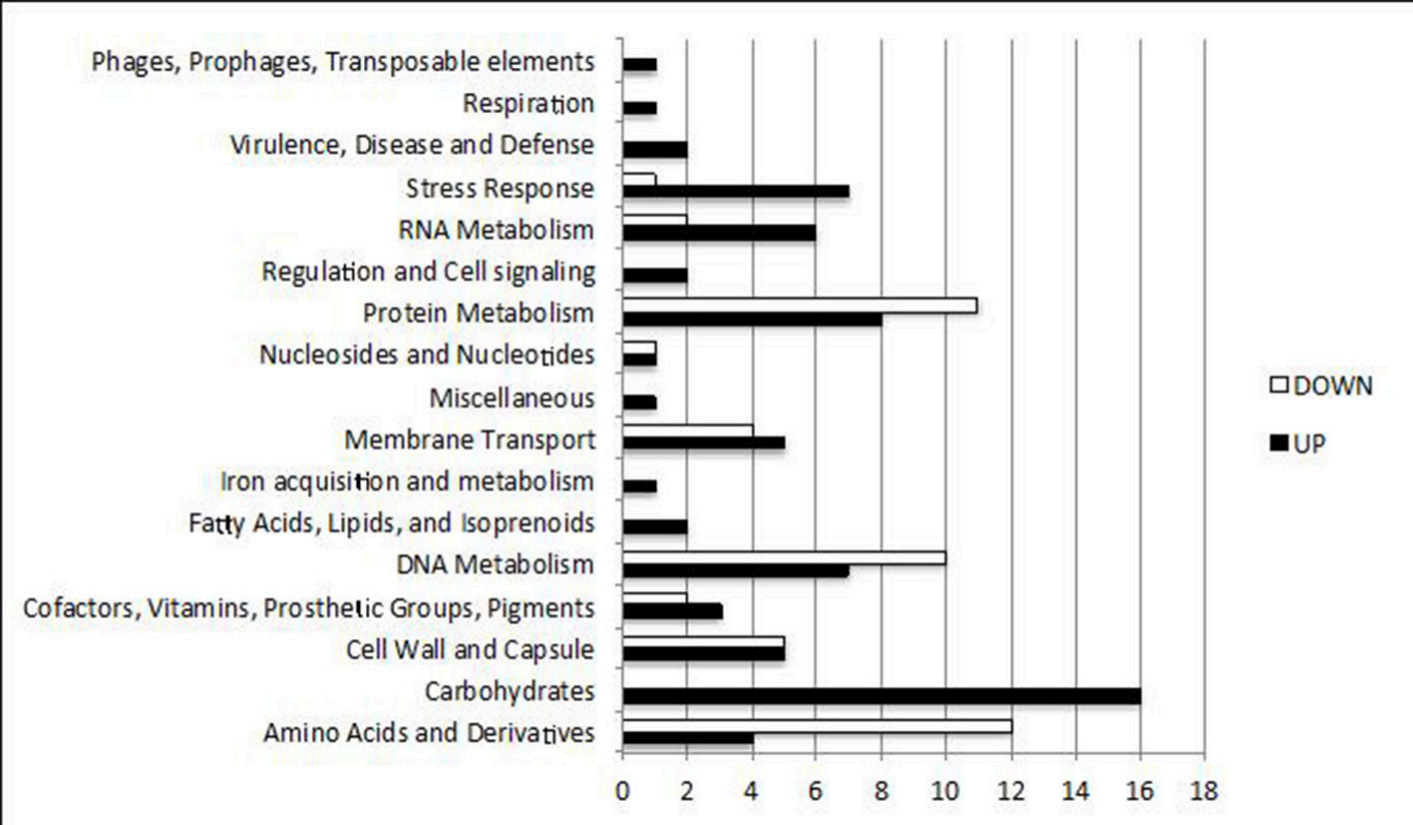
Functional categories differentially expressed in the two strains. Categories enriched with genes significantly up-regulated (black bars) and down-regulated (white bars) in TH1436 (Gal^+^) compared to TH1477 (Gal^−^). Categories were defined according to the SEED subsystems.

The overall transcriptional comparison between *S. thermophilus* TH1436 (Gal^+^) and TH1477 (Gal^−^) showed 133 genes significantly differentially expressed. Considering the Gal^+^ strain, 82 genes were higher and 51 lower expressed compared to the Gal^−^ strain (Table S1). Functional annotation was present for 116 transcripts of the total differentially expressed, among which 83 were automatically annotated and 33 manually refined and assigned to SEED first level categories. It was not possible to produce any annotation for the remaining 17 transcripts. The “Carbohydrate” category was the class with the highest number (14) of statistically significantly highly expressed genes in the Gal^+^ strain (Figure 4), particularly the “Di-,” “Oli-,” and “Mono- saccharides” subcategory (Table S2). Other statistically different categories were “DNA replication” and “Stress response” (Table S2, Figure 4), while the category with the highest number of low expressed genes was “Amino acids and derivatives.” Interestingly, genes related to “DNA metabolism” (Figure 4) and in particular several CRISPRs associated proteins were also expressed at a lower level in the Gal^+^ strain (Table 4). Overall considered, these findings indicate that significant increase in carbohydrate metabolism and in stress response occurred in the Gal^+^ strain, while DNA, protein and amino acids metabolism were negatively regulated (Figure 4).

**Table 4:** Selection of genes differentially expressed in strains TH1436 (Gal^+^) and TH1477 (Gal^−^).

**RPKM**							
**TH1436**	**TH1477**	**Fold change**	***p*-value**	**Description**	**Gene ID**	**EC**	**Category**
223 ± 77	43 ± 9	8.62	***0.000	Phage shock protein C	gene_0630		Stress response
2459 ± 285	489 ± 161	8.56	***0.000	PTS system, sucrose-specific IIA; IIB; IIC component	gene_1503	2.7.1.69	Carbohydrates
1489 ± 398	344 ± 86	7.38	***0.000	Superoxide dismutase, DNA binding protein	gene_1775	1.15.1.1	Stress response
1895 ± 231	573 ± 393	6.50	***0.000	Phage infection protein	gene_1732		Stress response
2959 ± 215	669 ± 73	6.43	***0.000	Galactose-1-phosphate uridylyltransferase	gene_1307	2.7.7.10	Carbohydrates
4759 ± 841	1015 ± 430	6.33	***0.000	Galactokinase	gene_1308	2.7.1.6	Carbohydrates
518 ± 115	176 ± 63	5.19	***0.000	Sucrose operon repressor ScrR	gene_1505		Carbohydrates
994 ± 138	351 ± 173	5.17	***0.000	Acetate kinase	gene_1765	2.7.2.1	Carbohydrates
2047 ± 93	615 ± 32	4.92	***0.000	UDP-glucose 4-epimerase	gene_1306	5.1.3.2	Cell wall and capsule
1195 ± 14	484 ± 368	4.88	***0.000	2',3'-cyclic-nucleotide 2'-phosphodiesterase	gene_0286	3.1.4.16	Nucleosides and nucleotides
1132 ± 39	394 ± 50	4.33	***0.001	PTS system, fructose-specificIIA; IIB; IIC component	gene_0394	2.7.1.69	Carbohydrates
295 ± 42	115 ± 32	4.22	***0.000	Fumarate reductase, flavoprotein subunit precursor	gene_1676	1.3.99.1	Respiration
181 ± 29	88 ± 13	3.33	***0.000	Acetoin utilization acuB protein	gene_0331	4.2.1.11	Carbohydrates
31 ± 9	162 ± 56	−4.13	***0.000	Response regulator SaeR	gene_1264		Cell wall and capsule
1985 ± 402	630 ± 176	4.48	**0.002	Catabolite control protein A	gene_0619		Regulation and cell signaling
883 ± 250	297 ± 82	4.29	**0.002	GTP-sensing transcriptional pleiotropic repressor codY	gene_1392		Stress response
873 ± 163	386 ± 89	3.27	**0.009	Phosphate acetyltransferase	gene_1623	2.3.1.8	Carbohydrates
811 ± 18	405 ± 76	3.18	**0.003	Phage lysin, glycosyl hydrolase, family 25	gene_0686		Phages, prophages, transposable elements
549 ± 94	378 ± 211	2.53	**0.009	Glutathione reductase	gene_0395	1.8.1.7	Stress response
210 ± 51	151 ± 62	2.27	**0.002	D-alanyl-D-alanine carboxypeptidase	gene_0094	3.4.16.4	Cell wall and capsule
53 ± 27	178 ± 18	−2.42	**0.004	CRISPR-associated RAMP Csm3	gene_0975		DNA metabolism
86 ± 38	309 ± 3	−2.66	**0.002	CRISPR-associated protein, Csm5 family	gene_0978		DNA metabolism
39 ± 27	156 ± 20	−2.94	**0.002	CRISPR repeat RNA endoribonuclease Cas6	gene_0971		DNA metabolism
4352 ± 678	1743 ± 641	3.42	*0.012	Pyruvate formate-lyase	gene_1419	2.3.1.54	Carbohydrates
4234 ± 764	1669 ± 965	3.28	*0.024	Heat shock protein GrpE	gene_0097		Protein metabolism
40253 ± 8829	16815 ± 10804	3.07	*0.041	Beta-galactosidase	gene_1302	3.2.1.23	Carbohydrates
4501 ± 325	1993 ± 1046	2.89	*0.036	1-phosphofructokinase	gene_0392	2.7.1.56	Carbohydrates
22508 ± 3470	10029 ± 6199	2.86	*0.050	Chaperone protein DnaK	gene_0098		Protein metabolism
563 ± 134	281 ± 117	2.75	*0.034	Universal stress protein family	gene_1395		Stress response
177 ± 19	157 ± 73	1.99	*0.017	Peptidoglycan N-acetylglucosamine deacetylase	gene_1316	3.5.1	Cell wall and capsule
223 ± 52	202 ± 87	1.80	*0.027	PTS system, mannose-specific IIC component	gene_0340	2.7.1.69	Carbohydrates
86 ± 40	228 ± 95	−1.70	*0.050	CRISPR-associated protein Cas1	gene_0969		DNA metabolism
160 ± 63	423 ± 81	−1.82	*0.039	Urea channel UreI	gene_0146		Amino acids and derivatives
103 ± 51	258 ± 19	−1.91	*0.040	Urease accessory protein UreD	gene_0154		Amino acids and derivatives
61 ± 18	167 ± 17	−2.02	*0.033	Urease accessory protein UreE	gene_0151		Amino acids and derivatives
63 ± 43	172 ± 30	−2.03	*0.028	Urease accessory protein UreG	gene_0153		Amino acids and derivatives
45 ± 29	131 ± 16	−2.11	*0.028	CRISPR-associated RAMP protein, Csm4 family	gene_0977		DNA metabolism
244 ± 158	721 ± 100	−2.19	*0.033	CRISPR-associated protein, Csm1 family	gene_0972		DNA metabolism
246 ± 62	853 ± 377	−2.81	*0.024	Urease accessory protein UreF	gene_0152		Amino acids and derivatives

*Average values ± standard deviation of reads per kb per million mapped reads (RPKM) for each strain. Genes are assigned to three groups according to the level of statistical significance, which was reported with asterisks and p-values. The gene expression differences between TH1436 and TH1477 are expressed as fold change; positive values are referred to genes more expressed in TH1436, conversely negative values are those more expressed in TH1477. The description of each gene differentially expressed, the Gene ID and the Enzyme Commission (EC) number are indicated*.

### Carbohydrates metabolism and sugar transport

**Figure 5:**
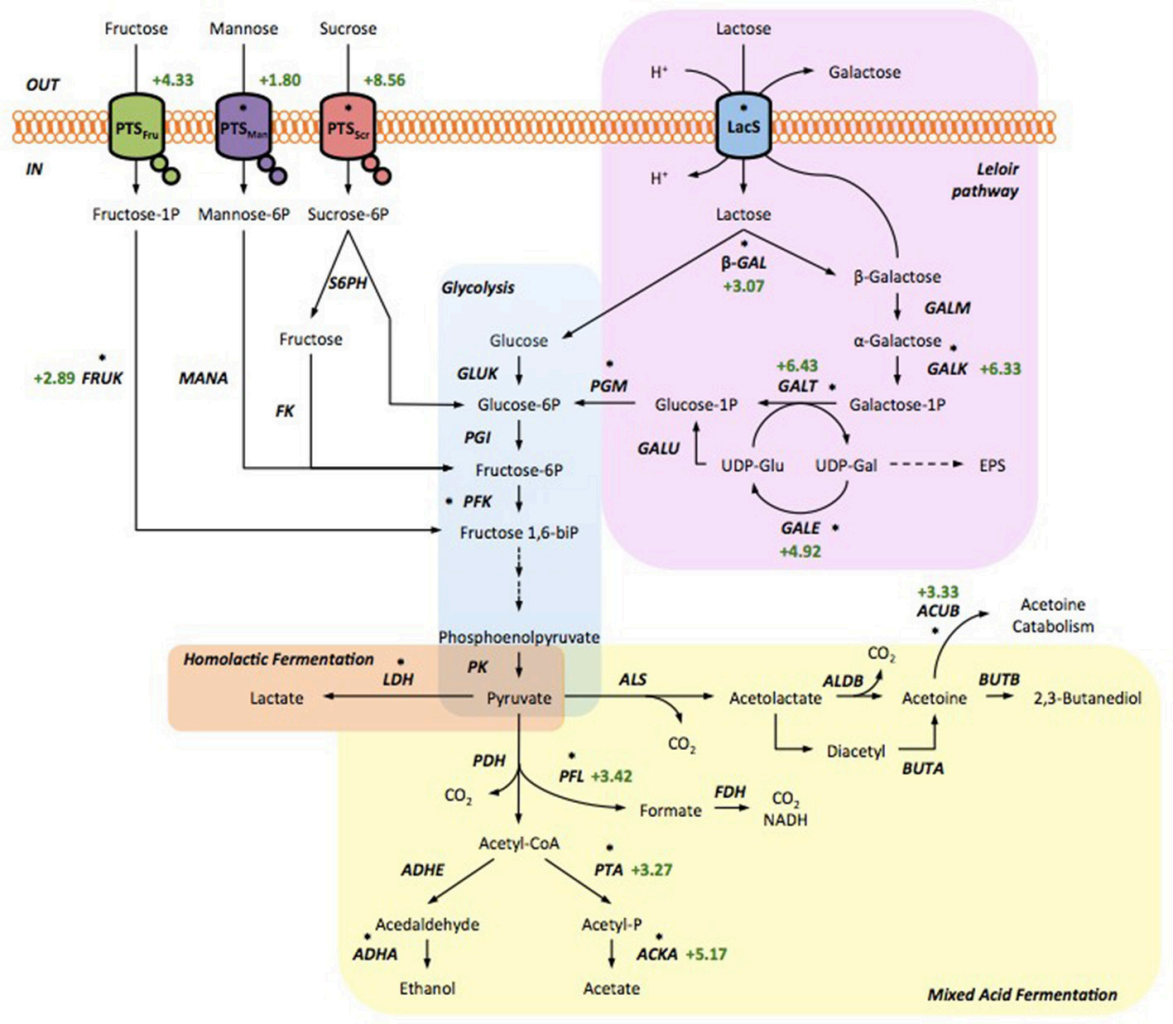
Schematic representation of main sugars metabolism with indication of differentially expressed genes. PTS transport systems (*PTS Scr*, PTS system sucrose-specific IIA; IIB; IIC component; *PTS Fru*, PTS system fructose-specific IIA; IIB; IIC component; *PTS Man*, PTS system mannose-specific IIA; IIB; IIC component). Lactose transport system (*LacS*, lactose permease; β*-GAL*, beta-galactosidase) and metabolism by the *Leloir pathway* (pink box; *GALM*, galactose mutarotase; *GALK*, galactokinase; *GALT*, galactose-1-phosphate uridylyltransferase; *GALE*, UDP-glucose 4-epimerase; *GALU*, UDP glucose pyrophosphorylase). Sugars are internalized (*S6PH*, sucrose-6-phosphate hydrolase; *FK*, fructokinase; *FRUK*, 1-phosphofructokinase; *MANA*, mannose 6-phosphate isomerase; *PGM*, phosphoglucomutase) in the Embden-Meyerhof-Parnas pathway (*Glycolysis*, light blue box; *GLUK*, glucose kinase; *PGI*, phosphoglucose isomerase; *PFK*, phosphofructokinase; *PK*, pyruvate kinase). Pyruvate is converted to lactate by *Homolactic Fermentation* (orange box; *LDH*, lactate dehydrogenase) or metabolized to other products by the *Mixed Acid Fermentation* (yellow box; *PDH*, pyruvate dehydrogenase; *PFL*, pyruvate formate-lyase; *FDH*, formate dehydrogenase; *PTA*, phosphate acetyltransferase; *ACKA*, acetate kinase; *ADHE*, acetaldehyde-CoA/alcohol dehydrogenase; *ADHA*, alcohol dehydrogenase; *ALS*, acetolactate synthase; *ALDB*, alpha-acetolactate decarboxylase; *BUTA*, acetoin reductase; *BUTB*, 2,3-butanediol dehydrogenase; *ACUB*, acetoin utilization protein). The genes regulated by CcpA are marked with an asterisk. Numbers represent differences in expression determined with RNA-seq.

Inside this category, the genes with the highest expression levels are related to galactose metabolism (Figure 5). In particular, the genes of the *gal* operon, namely *galK, galT*, and *galE* were significantly expressed more than 5-fold in the Gal^+^ (Table 4). This result strongly suggests that the expression of *galKTE* cluster is crucial to confer the ability to use galactose to *S. thermophilus*. This outcome validates the hypothesis from previous studies (Vaughan et al., 2001; van den Bogaard et al., 2004), that SNPs found in the *galK* promoter might have important effect on *galKTE* expression. The remaining *gal* genes, aldose 1-epimerase/galactose mutarotase (GalM; EC 5.1.3.3) and the regulatory protein GalR were not significantly differentially expressed.

Additionally, it is known that enzymes of the Leloir pathway can be involved in EPS production, since UDP-glucose and UDP-galactose are EPS precursors (Levander and Svensson, 2002). Indeed, the Gal^+^ strain produces greater amounts of EPS (Vendramin et al., 2017) suggesting that the use of Gal^+^ strains could be advantageous in technological processes to improve quality and texture of dairy products.

Regarding the *lac* operon genes, beta-galactosidase (LacZ, EC 3.2.1.23) was found 3-fold more expressed in the Gal^+^, suggesting a faster rate of lactose cleavage when galactose is metabolized.

No alterations of the promoter and gene sequence were found in *lacZ* (see Figures S1, S2 online), therefore this change in expression might be due to changes in activity of its transcriptional regulator.

Another finding is related to the phosphotransferase system (PTS) for sucrose and fructose, found to be more expressed (8.5- and 4.3-fold respectively) in the Gal^+^ strain. Fructose and glucose are poorly metabolized sugars, whereas sucrose is the preferred carbon source for *S. thermophilus*, besides lactose (van den Bogaard et al., 2000, 2004; Gunnewijk et al., 2001). However, Gal^+^ strains show the ability to utilize fructose as energy source, contrary to Gal^−^ strains (van den Bogaard et al., 2004). On the other hand, a link between sucrose PTS system with LacS has been reported and it has been shown that sucrose and lactose are used simultaneously and not in succession (Gunnewijk et al., 2001). Additionally, evidences suggest that the sucrose PTS system modulates lactose uptake by means of HPr during the stationary phase (Poolman et al., 1995). However, in our study lactose is the only sugar present in M17lac medium. The elevate expression of the sucrose and fructose PTS system recorded in the Gal^+^ strain could be related to the attempt of the starved cell to look outside for alternative energy sources (Thompson, 1987). The reason why this happened much less in the Gal^−^ strain needs further investigations. It could be hypothesized a repression by galactose, present in the medium of the Gal^−^ strain, since it is known that in *Streptococcus* the sucrose transporter is inhibited by lactose (Slee and Tanzer, 1979), which contains galactose.

Pathways related to pyruvate metabolism and involved in acetate production resulted more expressed in the Gal^+^ strain (Figure 5). The expression levels of phosphate acetyltransferase (*pta*) and acetate kinase (*acka*) were 3- and 5-fold higher in the Gal^+^, respectively. Moreover, pyruvate formate-lyase (*pfl*), acetoin utilization protein (*AcuB*), and flavoprotein subunit precursor of fumarate reductase were significantly more expressed (Table 4). Homolactic fermentation, in which pyruvate is converted entirely to lactate by lactate dehydrogenase (*ldh*), is the dominant sugar metabolism in *S. thermophilus*. However, it has been reported that, besides lactate, *S. thermophilus* produces low levels of secondary products such as acetate, α-acetolactate, acetoin (Teraguchi et al., 1987), acetaldehyde (Ott et al., 2000), and formate (Perez et al., 1991; Derzelle et al., 2005) by additional pyruvate dissipating pathways which were defined *in silico* (Hols et al., 2005). Homolactic fermentation is the most active energetic metabolic pathway in *Streptococcus* under anaerobic conditions in the presence of sugars. However, when environmental conditions change, e.g., reduced glycolytic flux (Loubiere et al., 1997), aerobic conditions (Gaudu et al., 2003) or change of carbon source (Thomas et al., 1980), *S. thermophilus* can switch to mixed-acid fermentation to produce ATP. The overexpression of genes involved in mixed acid fermentation suggests that the Gal^+^ strain after 18 h in M17lac shifted its energetic metabolism toward a mixed acid fermentation. Beside the enzymes and proteins involved directly in the metabolic pathway, it is important to highlight the relevance of regulatory proteins, in particular those involved in carbon catabolite repression (CCR), such as the pleiotropic transcriptional regulator CcpA. This protein is relevant for carbohydrate metabolism, in order to utilize efficiently the carbon source and to have the best energy profit under changing environmental conditions (Görke and Stülke, 2008). In the Gal^+^ strain CcpA expression was 4.48-fold higher than in the Gal^−^ suggesting that a different regulation is occurring. It has been demonstrated in *S. thermophilus* (van den Bogaard et al., 2000) and in *Lactococcus lactis* (Luesink et al., 1998) that CcpA activates the key genes of glycolysis [phosphofructokinase (*pfk*), piruvate kinase (*pk*)] and *ldh*, while it represses the *lac* operon and its own transcription. Genes under CcpA control have a *cre* sequence in their promoter region recognized and bound by CcpA. Therefore, we considered interesting to look for the presence of *cre* sites in the genome of TH1436 (Gal^+^) and TH1477 (Gal^−^) to find out which genes are controlled by CcpA. Surprisingly, beside 23 genes in common, the two strains showed a distinct series of genes controlled by CcpA, i.e., 28 genes for the Gal^+^ and 24 for the Gal^−^ (Table S3), including the genes of the *gal* operon (*galKTE*), suggesting that CcpA might lead the different regulation of the *gal* operon expression between the Gal^−^ and Gal^+^. According to this prediction, there were genes differentially expressed in the Gal^+^ strain (Table S1): the gal operon (*galKTE*), two PTS system specific for sucrose and mannose, four genes involved in mixed acid fermentation (*pfl, pta, acka*, and *acuB*), one involved in fructose metabolism (*fruK*) and *ccpA* itself (Figure 5). This indicates that the remodeling of carbohydrate metabolism in the Gal^+^ strain after 18 h of growth is likely due to CcpA regulation with the aim of maximizing the energy gain. However, a similar shift toward mixed acid fermentation recorded in the Gal^+^ strain had also been observed in *S. pneumoniae*, not as direct effect of CcpA positive regulation but rather as indirect effect by a galactose-induced alleviation of CcpA repression, indicating a more complex regulatory scenario when galactose is used as energy source (Carvalho et al., 2011). Indeed, experiments on CcpA transcriptional regulation of the *lac* operon of *S. thermophilus* strains evidenced that CcpA repression did not occur when cells grow on galactose in contrast to those grown on lactose. The same regulation occurred for *ldh* induction, although in the opposite manner, i.e., higher expression during growth on lactose and reduced on galactose (van den Bogaard et al., 2000).

Overall, in the present study the metabolism of the galactose moiety in the Gal^+^ strain through the Leloir pathway appears to reduce the contribution of glucose to the glycolytic flux. Indeed the lactose consumption at t6 revealed a faster decrease of lactose in the Gal^−^ strain, that take advantage of lactose/galactose antiporter system, which influences the rate of lactose transport into the cell. Moreover, it is expected that the production rate of Glu-1P in the Leloir pathway (which is a multi-step process) is slower than those originated by the lactose cleavage. The reduced glycolytic flux leads to the reduction of CCR, probably by low FBP intracellular concentration. The reduced action of CCR then determines a relaxed repression of *lac* and PTS (fructose and sucrose) operons expression and a reduced induction of *ldh*, thus deviating carbon metabolism from homolactic to mixed-acid fermentation. In contrast, in the metabolism of the Gal^−^ strain only glucose, derived from lactose, is metabolized, generating a high rate of glycolytic flux, that in turn triggers CCR thus leading carbon metabolism toward the homolactic fermentation. The same CCR alleviation that occurred in Gal^+^ strains, might happen even for some Gal^−^ strains when they start to re-internalize galactose after lactose exhaustion, a circumstance that was reported by other authors (Vaillancourt et al., 2002; Vin et al., 2005).

The *cre* site prediction indicates that not only genes related to carbohydrate metabolism, but also a series of genes involved in amino acids metabolism (alanine dehydrogenase, aspartate-semialdehyde dehydrogenase) and in stress defense response (i.e., NADH peroxidase, GTPase involved in stress response) are under the CcpA control (Table S3). This indication was also confirmed by the expression changes in specific categories of the Gal^+^ transcriptome (Figure 4). These findings show that CcpA can influence several cellular processes, like nitrogen metabolism and stress response, which concurrently with carbohydrates metabolism contribute to overcome environmental changes and succeed in getting nutrients (Görke and Stülke, 2008). This is in accordance with what was also found in the *S. pneumoniae* transcriptome where CcpA showed a strong impact on amino acids metabolism and on virulence factors (i.e., cell wall and capsule synthesis genes) in order to provide optimal metabolic adaptation (Carvalho et al., 2011).

### Energy consuming metabolism

The genes having a reduced expression in the Gal^+^ strain (assigned to “Amino acids and derivatives” and “DNA metabolism” categories) (Figure 4) together with the assumptions made from *cre* site prediction are consistent with the hypothesis that a partial reduction of energy-consuming processes is occurring in the Gal^+^ strain. A previous proteomic analysis performed on *S. thermophilus* under lactose starvation evidenced a downregulation of enzymes involved in glycolysis and protein biosynthesis. On the other hand, an up-regulation of enzymes involved in lactose (LacZ) and galactose catabolism (GalU, GalE and GalM), amino acid import and stress response was detected (Arena et al., 2006). A similar scenario was found in our transcriptomic analysis in which, at the stationary phase, the Gal^+^ strain had *lacZ*, “Leloir pathway” and “stress response” genes highly expressed while “proteins,” “amino acids,” and “DNA metabolism” genes were less expressed. In particular, four urease accessory proteins and urea channel in the Gal^+^ strain were found less expressed. *S. thermophilus* is the only lactic acid bacterium used as starter culture that possesses urease activity and urease negative strains are rare in nature (Monnet et al., 2004; Hols et al., 2005). It has been reported that in *S. thermophilus* and in *S. salivarius* a low pH induces urease genes expression when the culture is in nitrogen limited conditions (Arioli et al., 2007) to provide nitrogen for the synthesis of amino acids, such as glutamine (Monnet et al., 2005). The low expression level of urease could indicate a reduction in the need of ammonia in the Gal^+^ strain, suggesting an energy saving behavior by reduction of some cellular biosynthetic processes, such as amino acid metabolism.

In addition, six CRISPR associated proteins were found less expressed in the Gal^+^ strain by 2-fold: two belonging to the Cas family proteins (Cas1 and Cas6), and 4 members of Csm family proteins (Csm1, Csm3, Csm4, Csm5). Nowadays, *S. thermophilus* is considered a CRISPR-model in adaptation and evolution studies (Deveau et al., 2010; Rath et al., 2015). Proteomic studies in *S. thermophilus* revealed that most Cas proteins are constitutively expressed (Young et al., 2012), thus determining high energetic costs (Vale et al., 2015). Hence, the low expression of several CRISPR-associated proteins detected in the Gal^+^ strain could indicate an additional energy saving response from non-essential DNA processes (CRISPR and DNA repair). In our study, the energetic demand in the Gal^+^ strain occurred earlier than in the Gal^−^ during growth and this is significant not only in the fermentation metabolism but also in the reduction of biosynthetic processes in to save energy.

### Stress response

Since at the point when the transcriptomic analysis was performed bacteria were living in stressful conditions, mainly for low pH and absence of energy source, we thought interesting to examine the different expression of genes related to stress response in the two strains. A particularly interesting finding is the presence of a putative DNA binding protein, manually annotated as a superoxide dismutase (SOD), that was 7.38-fold more expressed in the Gal^+^ strain (Table 4). *S. thermophilus* is an aerotolerant microorganism that can tolerate the presence of oxygen because of its antioxidant defense systems (Thibessard et al., 2001, 2004; Marco et al., 2013; Zhang et al., 2015). A manganese-dependent superoxide dismutase (MnSOD) was previously found expressed at considerably high levels in *S. thermophilus* during the early stationary phase (Chang and Hassan, 1997) and it has been suggested its protective role not only against oxidative stress but also against acidic stress due to the production of lactic acid during fermentation (Bruno-Bárcena et al., 2010). At the time point of our transcriptional analysis sampling, bacterial growth was reduced, and lactic acid accumulated in the medium. During mixed acid fermentation, further organic acids were produced thus possibly triggering a stress response. Moreover, the starvation state of the Gal^+^ strain is supported by the fact that MnSOD was shown determinant also for a *Staphylococcus aureus* MnSOD mutant survival during long-term starvation, particularly during amino acid limitation and acidic stress (Clements et al., 1999).

In addition, a series of stress related genes were highly expressed, such as heat shock protein GrpE, chaperone protein DnaK, and glutathione reductase (Table 4). The latter enzyme was already found as stress defense response against ROS when *S. thermophilus* CNRZ368 strain was grown under aerobic conditions (Pébay et al., 1995). Moreover, a specific GTP and DNA binding protein (GTP-sensing transcriptional pleiotropic repressor CodY) was 4.29-fold more expressed in the Gal^+^ strain (Table 4). This protein works as molecular sensor of nutritional limitations by sensing the intracellular GTP concentration and regulates the expression of several genes involved in transition from exponential to stationary phase (Sonenshein, 1996; Ratnayake-Lecamwasam et al., 2001). Two “Stress response” genes were highly overexpressed in the Gal^+^ strain: phage shock protein C (PspC) (8.62-fold) and phage infection protein (Pip) (6.5-fold) (Table 4). Pip is an integral membrane protein that works as phage receptor which is required for phage infection in *L. lactis* (Mooney et al., 2006). PspC is one of the 6 members of the phage shock proteins system. These proteins together maintain membrane integrity and hence the proton motive force (PMF). It has been proposed that Psp are induced by the reduction of the energy status and dissipation of the PMF. In particular, PspC is a membrane protein able to sense alterations in the PMF and to induce the Psp system response (Darwin, 2005). Accordingly, several cell wall proteins were differently expressed, suggesting an alteration and remodeling of the cell wall and membrane in the Gal^+^ strain.

## Conclusion

In our work the most advanced transcriptomic profiling approaches were used to compare a Gal^+^ (TH1436) strain with a Gal^−^ (TH1477). This modern technology allowed a reliable detection of the *galKTE* genes differential expression, which in the Gal^+^ strain was 5–6-fold higher than in the Gal^−^, and strongly suggests that the main determinant of the Gal^−^ phenotype is the low level of the *gal* operon expression. The SNPs in the *cre* site together with the presence of the *galKTE* cluster in the list of predicted genes controlled by CcpA in the Gal^+^ strain only, encourages the hypothesis that CcpA might drive the different regulation of *galKTE* expression between Gal^−^ and Gal^+^ strains.

Overall, the transcriptomic scenario shows that, after 18 h of growth, the Gal^+^ strain underwent a deep metabolic remodeling. In particular, a shift from homolactic fermentation toward the energetically favorable mixed acid fermentation was evidenced. In addition, enhancement of several genes involved in stress response and changes in proteins and amino acids related metabolism indicate that the Gal^+^ strain is adopting a saving energy behavior. The influence of CcpA on gene regulation suggests that the Gal^+^ strain adopts different transcriptional regulations compared to the Gal^−^ to optimize its adaptation during the stationary phase.

It can be concluded that the Gal^+^ strain differs from the Gal^−^ not only in galactose metabolism but also in operation and regulation of other metabolic pathways. This could evidently be ascribed in part to the different genetic background of the two strains, but anyway our work evidenced the different response of all the genes that the two strains have in common, during their growth in a complex medium having lactose as the most abundant energy source.

Since this is the first time that a transcriptomic approach is used to study the gal/lac catabolism, it could represent a helpful reference for further analyses of other strains. The possibility to increase our knowledge of Gal^+^ strains metabolism is of enormous interest for the dairy industry, which could implement technological processes for increasing the quality of dairy products and limiting the negative impacts of galactose accumulation.

## Author contributions

SG drafted the manuscript, interpreted gene expression results, performed Gal operon sequence inspection. SG and LT performed the regulatory site prediction analyses. SG and AT performed lactose and galactose consumption analyses. LT, VC, and AG conceived and designed the experiment. LT and VV performed RNA extraction. VV performed the acidification curves. LT did bioinformatics sequencing analyses and differential gene expression calculation. VSD was involved in genetic and sequence analyses, data interpretation, and drafting of the manuscript. AT performed growth evaluation on galactose. SC performed the transcriptome profile reconstruction and the statistical analyses regarding SEED functional categories and differentially expressed genes. VC participated in discussion of paper writing. VC, AG, LT, and SC revised the manuscript.

### Conflict of interest statement

The authors declare that the research was conducted in the absence of any commercial or financial relationships that could be construed as a potential conflict of interest.
