# Supplementary material for: Comparative Transcriptomic Analysis of Streptococcus thermophilus TH1436 and TH1477 Showing Different Capability in the Use of Galactose
Source: Front Microbiol. 2018 Aug 7;9:1765. doi: 10.3389/fmicb.2018.01765 (PMC6090898; doi:10.3389/fmicb.2018.01765)
Supplement: Supplementary file 1 [file Table_1.docx]

Table S1 Genes differentially expressed in TH1436 (Gal^+^) and TH1477 (Gal^-^) strains

Average values ± standard deviation of reads per kb per million mapped reads (RPKM) for each strain. Three groups are made according to the level of statistical significance, which was indicated with asterisks and p-value. The gene expression differences between TH1436 and TH1477 are expressed as fold change; positive values are referred to genes more expressed in TH1436, conversely negative values are those more expressed in TH1477. Description of each gene differentially expressed, the Gene ID and the Enzyme Commission (EC) number are indicated.

| RPKM | | | | | |  |  |  |  |  |  |  |
| --- | --- | --- | --- | --- | --- | --- | --- | --- | --- | --- | --- | --- |
| TH1436 | | | TH1477 | | | Fold change | p-value | | Description | Gene ID | EC | Category |
| 223 | ± | 77 | 43 | ± | 9 | 8.62 | *** | 0.000 | Phage shock protein C | gene_0630 |  | Stress Response |
| 2459 | ± | 285 | 489 | ± | 161 | 8.56 | *** | 0.000 | PTS system, sucrose-specific IIA; IIB; IIC component | gene_1503 | 2.7.1.69 | Carbohydrates |
| 1489 | ± | 398 | 344 | ± | 86 | 7.38 | *** | 0.000 | Superoxide dismutase, DNA binding protein | gene_1775 | 1.15.1.1 | Stress Response |
| 1895 | ± | 231 | 573 | ± | 393 | 6.50 | *** | 0.000 | Phage infection protein | gene_1732 |  | Stress Response |
| 2959 | ± | 215 | 669 | ± | 73 | 6.43 | *** | 0.000 | Galactose-1-phosphate uridylyltransferase | gene_1307 | 2.7.7.10 | Carbohydrates |
| 4759 | ± | 841 | 1015 | ± | 430 | 6.33 | *** | 0.000 | Galactokinase | gene_1308 | 2.7.1.6 | Carbohydrates |
| 518 | ± | 115 | 176 | ± | 63 | 5.19 | *** | 0.000 | Sucrose operon repressor (ScrR) | gene_1505 | 4.2.1.11 | Carbohydrates |
| 994 | ± | 138 | 351 | ± | 173 | 5.17 | *** | 0.000 | Acetate kinase | gene_1765 | 2.7.2.1 | Carbohydrates |
| 480 | ± | 63 | 161 | ± | 56 | 5.14 | *** | 0.000 | Nucleotidyltransferase | gene_0373 | 2.7.7 | RNA Metabolism |
| 50 | ± | 14 | 18 | ± | 9 | 5.03 | *** | 0.000 | hypothetical protein | gene_1630 |  | ND |
| 2047 | ± | 93 | 615 | ± | 32 | 4.92 | *** | 0.000 | UDP-glucose 4-epimerase | gene_1306 | 5.1.3.2 | Cell Wall and Capsule |
| 1061 | ± | 310 | 336 | ± | 37 | 4.89 | *** | 0.001 | Lipoate-protein ligase A | gene_1024 | 2.7.7.63 | Cofactors, Vitamins, Prosthetic Groups, Pigments |
| 1195 | ± | 14 | 484 | ± | 368 | 4.88 | *** | 0.000 | 2',3'-cyclic-nucleotide 2'-phosphodiesterase | gene_0286 | 3.1.4.16 | Nucleosides and Nucleotides |
| 198 | ± | 66 | 73 | ± | 5 | 4.33 | *** | 0.000 | Acetoin utilization protein AcuB | gene_0332 | 4.2.1.11 | Carbohydrates |
| 1132 | ± | 39 | 394 | ± | 50 | 4.33 | *** | 0.001 | PTS system, fructose-specificIIA; IIB; IIC component | gene_0394 | 2.7.1.69 | Carbohydrates |
| 295 | ± | 42 | 115 | ± | 32 | 4.22 | *** | 0.000 | Fumarate reductase, flavoprotein subunit precursor | gene_1676 | 1.3.99.1 | Respiration |
| 181 | ± | 29 | 88 | ± | 13 | 3.33 | *** | 0.000 | Acetoin utilization acuB protein | gene_0331 | 4.2.1.11 | Carbohydrates |
| 40 | ± | 23 | 175 | ± | 25 | -3.46 | *** | 0.001 | Valyl-tRNA synthetase | gene_0681 | 6.1.1.9 | Protein Metabolism |
| 31 | ± | 9 | 162 | ± | 56 | -4.13 | *** | 0.000 | Response regulator SaeR | gene_1264 | 6.3.2.4 | Cell Wall and Capsule |
| 74 | ± | 32 | 459 | ± | 184 | -5.06 | *** | 0.000 | Prolyl-tRNA synthetase | gene_0537 | 6.1.1.15 | Protein Metabolism |
| 92 | ± | 72 | 623 | ± | 191 | -5.93 | *** | 0.000 | LysM-Peptidoglycan-binding protein | gene_0680 |  | Cell Wall and Capsule |
| 211 | ± | 45 | 1662 | ± | 976 | -6.63 | *** | 0.000 | tRNA-dependent lipid II-AlaAla--L-alanine ligase | gene_0310 |  | Cell Wall and Capsule |
| 1813 | ± | 885 | 536 | ± | 271 | 4.74 | ** | 0.006 | Acyl carrier protein | gene_0357 |  | Fatty Acids, Lipids, and Isoprenoids |
| 1985 | ± | 402 | 630 | ± | 176 | 4.48 | ** | 0.002 | Catabolite control protein A | gene_0619 |  | Regulation and Cell signaling |
| 883 | ± | 250 | 297 | ± | 82 | 4.29 | ** | 0.002 | GTP-sensing transcriptional pleiotropic repressor codY | gene_1392 |  | Stress Response |
| 371 | ± | 125 | 131 | ± | 52 | 4.04 | ** | 0.002 | FIG01115437: hypothetical protein | gene_0467 |  | ND |
| 82 | ± | 52 | 40 | ± | 36 | 3.53 | ** | 0.003 | Mobile element protein | gene_1811 |  | ND |
| 873 | ± | 163 | 386 | ± | 89 | 3.27 | ** | 0.009 | Phosphate acetyltransferase | gene_1623 | 2.3.1.8 | Carbohydrates |
| 517 | ± | 78 | 308 | ± | 233 | 3.18 | ** | 0.003 | L-asparaginase | gene_1490 | 3.5.1.1 | Amino Acids and Derivatives |
| 811 | ± | 18 | 405 | ± | 76 | 3.18 | ** | 0.003 | Phage lysin, glycosyl hydrolase, family 25 | gene_0686 |  | Phages, Prophages, Transposable elements |
| 27 | ± | 2 | 16 | ± | 9 | 3.05 | ** | 0.004 | FIG01116906: hypothetical protein | gene_1531 |  | ND |
| 638 | ± | 4 | 336 | ± | 46 | 2.93 | ** | 0.006 | Ribonuclease BN | gene_1638 | 3.1 | RNA Metabolism |
| 690 | ± | 14 | 358 | ± | 20 | 2.89 | ** | 0.008 | Phosphopantetheine adenylyltransferase | gene_1409 | 2.7.7.3 | Cofactors, Vitamins, Prosthetic Groups, Pigments |
| 823 | ± | 36 | 455 | ± | 89 | 2.87 | ** | 0.007 | GTP-binding protein EngA | gene_0189 | 3.1.26.12 | Protein Metabolism |
| 494 | ± | 72 | 286 | ± | 63 | 2.85 | ** | 0.006 | DNA polymerase III beta subunit | gene_0005 | 2.7.7.7 | DNA Metabolism |
| 536 | ± | 39 | 308 | ± | 68 | 2.83 | ** | 0.006 | Proline dipeptidase | gene_0618 | 3.4.13.9 | Amino Acids and Derivatives |
| 252 | ± | 41 | 152 | ± | 22 | 2.68 | ** | 0.003 | Iojap protein | gene_1374 | 3.1.26.12 | RNA Metabolism |
| 386 | ± | 60 | 222 | ± | 5 | 2.67 | ** | 0.006 | Amino acid ABC transporter, amino acid-binding protein | gene_1556 |  | Membrane Transport |
| 926 | ± | 104 | 578 | ± | 204 | 2.63 | ** | 0.010 | DNA polymerase I | gene_1532 | 2.7.7.7 | DNA Metabolism |
| 224 | ± | 60 | 140 | ± | 13 | 2.58 | ** | 0.006 | Transcriptional regulator, TetR family | gene_1733 |  | Amino Acids and Derivatives |
| 118 | ± | 21 | 91 | ± | 66 | 2.53 | ** | 0.007 | unknown protein | gene_1810 |  | ND |
| 549 | ± | 94 | 378 | ± | 211 | 2.53 | ** | 0.009 | Glutathione reductase | gene_0395 | 1.8.1.7 | Stress Response |
| 123 | ± | 28 | 92 | ± | 47 | 2.28 | ** | 0.004 | Amino acid ABC transporter, ATP-binding protein | gene_0793 |  | Membrane Transport |
| 210 | ± | 51 | 151 | ± | 62 | 2.27 | ** | 0.002 | D-alanyl-D-alanine carboxypeptidase | gene_0094 | 3.4.16.4 | Cell Wall and Capsule |
| 345 | ± | 36 | 267 | ± | 125 | 2.24 | ** | 0.007 | Manganese transport protein MntH | gene_0642 |  | Membrane Transport |
| 227 | ± | 20 | 154 | ± | 23 | 2.23 | ** | 0.008 | Nicotinate-nucleotide adenylyltransferase | gene_1376 | 2.7.7.18 | Cofactors, Vitamins, Prosthetic Groups, Pigments |
| 172 | ± | 35 | 143 | ± | 73 | 2.04 | ** | 0.008 | Acyl-phosphate:glycerol-3-phosphate O-acyltransferase PlsY | gene_0571 |  | Fatty Acids, Lipids, and Isoprenoids |
| 144 | ± | 40 | 533 | ± | 139 | -2.41 | ** | 0.003 | Serine hydroxymethyltransferase | gene_0712 | 2.1.2.1 | Cofactors, Vitamins, Prosthetic Groups, Pigments |
| 53 | ± | 27 | 178 | ± | 18 | -2.42 | ** | 0.004 | CRISPR-associated RAMP Csm3 | gene_0975 |  | DNA Metabolism |
| 95 | ± | 75 | 343 | ± | 80 | -2.64 | ** | 0.002 | Alpha-D-GlcNAc alpha-1,2-L-rhamnosyltransferase | gene_0868 | 2.4.1 | Cell Wall and Capsule |
| 86 | ± | 38 | 309 | ± | 3 | -2.66 | ** | 0.002 | CRISPR-associated protein, Csm5 family | gene_0978 |  | DNA Metabolism |
| 51 | ± | 25 | 184 | ± | 1 | -2.68 | ** | 0.003 | DNA-binding response regulator | gene_1265 |  | ND |
| 73 | ± | 43 | 267 | ± | 35 | -2.68 | ** | 0.001 | ABC transporter, ATP-binding protein | gene_1478 |  | Membrane Transport |
| 14 | ± | 9 | 55 | ± | 5 | -2.90 | ** | 0.007 | Phosphoribosyl-ATP pyrophosphatase | gene_1095 | 3.6.1.31 | Amino Acids and Derivatives |
| 39 | ± | 27 | 156 | ± | 20 | -2.94 | ** | 0.002 | CRISPR repeat RNA endoribonuclease Cas6 | gene_0971 |  | DNA Metabolism |
| 17 | ± | 15 | 89 | ± | 61 | -3.33 | ** | 0.005 | ABC transporter | gene_0141 |  | Membrane Transport |
| 12 | ± | 3 | 63 | ± | 11 | -3.91 | ** | 0.001 | oxidoreductase, putative | gene_1263 |  | ND |
| 1213 | ± | 473 | 504 | ± | 182 | 3.46 | * | 0.015 | Ribosomal subunit interface protein | gene_0337 | 3.1.26.12 | Protein Metabolism |
| 4352 | ± | 678 | 1743 | ± | 641 | 3.42 | * | 0.012 | Pyruvate formate-lyase | gene_1419 | 2.3.1.54 | Carbohydrates |
| 4234 | ± | 764 | 1669 | ± | 965 | 3.28 | * | 0.024 | Heat shock protein GrpE | gene_0097 | 3.1.26.12 | Protein Metabolism |
| 40253 | ± | 8829 | 16815 | ± | 10804 | 3.07 | * | 0.041 | Beta-galactosidase | gene_1302 | 3.2.1.23 | Carbohydrates |
| 5348 | ± | 872 | 2257 | ± | 1347 | 3.03 | * | 0.035 | Peptide methionine sulfoxide reductase MsrA / MsrB | gene_1244 | 1.8.4.11/12 | Protein Metabolism |
| 4501 | ± | 325 | 1993 | ± | 1046 | 2.89 | * | 0.036 | 1-phosphofructokinase | gene_0392 | 2.7.1.56 | Carbohydrates |
| 22508 | ± | 3470 | 10029 | ± | 6199 | 2.86 | * | 0.050 | Chaperone protein DnaK | gene_0098 | 3.1.26.12 | Protein Metabolism |
| 563 | ± | 134 | 281 | ± | 117 | 2.75 | * | 0.034 | Universal stress protein family | gene_1395 |  | Stress Response |
| 573 | ± | 160 | 319 | ± | 37 | 2.72 | * | 0.023 | Extracellular protein | gene_1151 |  | ND |
| 1214 | ± | 40 | 661 | ± | 29 | 2.71 | * | 0.017 | Putative deoxyribose-specific ABC transporter, ATP-binding protein | gene_0779 | 4.2.1.11 | Carbohydrates |
| 318 | ± | 90 | 175 | ± | 64 | 2.55 | * | 0.028 | FIG01115301: hypothetical protein | gene_0634 |  | ND |
| 41 | ± | 11 | 27 | ± | 4 | 2.53 | * | 0.023 | hypothetical protein | gene_1290 |  | ND |
| 95 | ± | 12 | 60 | ± | 3 | 2.44 | * | 0.014 | bacterial seryl-tRNA synthetase related | gene_0641 | 3.1.26.12 | Protein Metabolism |
| 870 | ± | 29 | 510 | ± | 49 | 2.44 | * | 0.032 | Putative deoxyribose-specific ABC transporter, permease protein | gene_0781 |  | Carbohydrates |
| 761 | ± | 125 | 449 | ± | 90 | 2.43 | * | 0.045 | ABC transporter ATP-binding protein | gene_1283 | 3.1.26.12 | Protein Metabolism |
| 73 | ± | 21 | 55 | ± | 37 | 2.43 | * | 0.016 | probably aromatic ring hydroxylating enzyme, evidenced by COGnitor; PaaD-like protein (DUF59) involved in Fe-S cluster assembly | gene_1358 |  | Miscellaneous |
| 520 | ± | 28 | 330 | ± | 26 | 2.40 | * | 0.023 | Putative deoxyribose-specific ABC transporter, permease protein | gene_0780 | 4.2.1.11 | Carbohydrates |
| 948 | ± | 123 | 647 | ± | 178 | 2.32 | * | 0.026 | Lon-like protease with PDZ domain | gene_1408 | 3.1.26.12 | Protein Metabolism |
| 506 | ± | 19 | 378 | ± | 171 | 2.31 | * | 0.019 | Chromosomal replication initiator protein DnaA | gene_0006 |  | DNA Metabolism |
| 415 | ± | 13 | 292 | ± | 71 | 2.28 | * | 0.011 | Helicase loader DnaI | gene_0188 |  | DNA Metabolism |
| 465 | ± | 17 | 327 | ± | 60 | 2.22 | * | 0.025 | Helicase loader DnaB | gene_0187 |  | DNA Metabolism |
| 242 | ± | 54 | 164 | ± | 23 | 2.20 | * | 0.031 | S-ribosylhomocysteine lyase / Autoinducer-2 production protein LuxS | gene_0386 | 4.4.1.21 | Amino Acids and Derivatives |
| 118 | ± | 9 | 81 | ± | 4 | 2.15 | * | 0.024 | ABC-type antimicrobial peptide transport system, permease component | gene_1282 |  | Virulence, Disease and Defense |
| 134 | ± | 12 | 109 | ± | 47 | 2.07 | * | 0.010 | MutT/NudX family protein (putative) | gene_1228 |  | ND |
| 411 | ± | 26 | 336 | ± | 134 | 2.06 | * | 0.022 | Adenine-specific methyltransferase | gene_1766 | 2.1.1.72 | DNA Metabolism |
| 275 | ± | 14 | 217 | ± | 62 | 2.05 | * | 0.014 | rRNA methylase | gene_0118 |  | RNA Metabolism |
| 441 | ± | 53 | 343 | ± | 88 | 2.04 | * | 0.030 | FIG01114336: hypothetical protein | gene_0402 |  | ND |
| 181 | ± | 17 | 160 | ± | 83 | 2.03 | * | 0.014 | LrgA-associated membrane protein LrgB | gene_1598 |  | Regulation and Cell signaling |
| 137 | ± | 10 | 121 | ± | 70 | 2.02 | * | 0.017 | Transposase | gene_1633 |  | DNA Metabolism |
| 83 | ± | 6 | 70 | ± | 29 | 2.01 | * | 0.029 | Na+ driven multidrug efflux pump | gene_0688 |  | Membrane Transport |
| 212 | ± | 22 | 163 | ± | 26 | 1.99 | * | 0.018 | Hydrolase (HAD superfamily), YqeK | gene_1375 |  | ND |
| 177 | ± | 19 | 157 | ± | 73 | 1.99 | * | 0.017 | Peptidoglycan N-acetylglucosamine deacetylase | gene_1316 | 3.5.1 | Cell Wall and Capsule |
| 345 | ± | 46 | 281 | ± | 89 | 1.98 | * | 0.021 | 16S rRNA (guanine(966)-N(2))-methyltransferase SSU rRNA m(2)G966 | gene_1411 | 2.1.1.171 | RNA Metabolism |
| 179 | ± | 35 | 155 | ± | 78 | 1.96 | * | 0.013 | tRNA pseudouridine synthase A | gene_0101 | 4.2.1.70 | Virulence, Disease and Defense |
| 189 | ± | 8 | 158 | ± | 37 | 1.91 | * | 0.025 | Hypothetical protein in cluster with Ecs transporter (in Streptococci) | gene_1366 |  | Membrane Transport |
| 226 | ± | 30 | 211 | ± | 128 | 1.91 | * | 0.023 | D-alanyl-D-alanine carboxypeptidase | gene_0060 | 3.4.16.4 | Cell Wall and Capsule |
| 130 | ± | 5 | 117 | ± | 47 | 1.87 | * | 0.029 | Substrate-specific component PdxU2 of predicted pyridoxin-related ECF transporter | gene_0103 |  | Iron acquisition and metabolism |
| 225 | ± | 28 | 201 | ± | 74 | 1.81 | * | 0.032 | rRNA small subunit methyltransferase I | gene_0355 |  | RNA Metabolism |
| 223 | ± | 52 | 202 | ± | 87 | 1.80 | * | 0.027 | PTS system, mannose-specific IIC component | gene_0340 | 2.7.1.69 | Carbohydrates |
| 129 | ± | 29 | 120 | ± | 52 | 1.78 | * | 0.034 | putative N-acetyl-muramidase | gene_0095 |  | Cell Wall and Capsule |
| 86 | ± | 40 | 228 | ± | 95 | -1.70 | * | 0.050 | CRISPR-associated protein Cas1 | gene_0969 |  | DNA Metabolism |
| 73 | ± | 26 | 208 | ± | 91 | -1.78 | * | 0.036 | Mobile element protein | gene_0089 |  | DNA Metabolism |
| 130 | ± | 99 | 337 | ± | 119 | -1.81 | * | 0.042 | 2-isopropylmalate synthase | gene_1065 | 2.3.3.13 | Amino Acids and Derivatives |
| 160 | ± | 63 | 423 | ± | 81 | -1.82 | * | 0.039 | Urea channel UreI | gene_0146 |  | Amino Acids and Derivatives |
| 203 | ± | 87 | 531 | ± | 74 | -1.83 | * | 0.047 | DNA repair protein RecN | gene_1074 |  | DNA Metabolism |
| 63 | ± | 11 | 174 | ± | 26 | -1.85 | * | 0.045 | putative Zn-dependent protease | gene_0953 | 3.1.26.12 | Protein Metabolism |
| 91 | ± | 44 | 260 | ± | 95 | -1.87 | * | 0.021 | N-acyl-L-amino acid amidohydrolase | gene_0167 | 3.5.1.14 | Protein Metabolism |
| 66 | ± | 45 | 192 | ± | 87 | -1.91 | * | 0.029 | Dihydrodipicolinate synthase | gene_1174 | 4.2.1.52 | Amino Acids and Derivatives |
| 103 | ± | 51 | 258 | ± | 19 | -1.91 | * | 0.040 | Urease accessory protein UreD | gene_0154 | 3.1.26.12 | Amino Acids and Derivatives |
| 109 | ± | 50 | 287 | ± | 8 | -1.93 | * | 0.030 | Transcription termination protein NusA | gene_0303 |  | RNA Metabolism |
| 91 | ± | 53 | 242 | ± | 33 | -1.94 | * | 0.024 | tmRNA-binding protein SmpB | gene_0615 | 3.1.26.12 | Protein Metabolism |
| 129 | ± | 30 | 357 | ± | 25 | -1.99 | * | 0.031 | S-adenosylmethionine synthetase | gene_0937 | 2.5.1.6 | Amino Acids and Derivatives |
| 58 | ± | 29 | 158 | ± | 6 | -2.01 | * | 0.031 | 3-isopropylmalate dehydratase small subunit | gene_1138 | 4.2.1.33 | Amino Acids and Derivatives |
| 61 | ± | 18 | 167 | ± | 17 | -2.02 | * | 0.033 | Urease accessory protein UreE | gene_0151 | 3.1.26.12 | Amino Acids and Derivatives |
| 63 | ± | 43 | 172 | ± | 30 | -2.03 | * | 0.028 | Urease accessory protein UreG | gene_0153 | 3.1.26.12 | Amino Acids and Derivatives |
| 70 | ± | 34 | 191 | ± | 1 | -2.04 | * | 0.023 | Pseudouridylate synthases, 23S RNA-specific | gene_0713 |  | Nucleosides and Nucleotides |
| 54 | ± | 21 | 152 | ± | 9 | -2.06 | * | 0.030 | Group B streptococcal surface immunogenic protein | gene_0431 |  | Stress Response |
| 40 | ± | 17 | 127 | ± | 43 | -2.08 | * | 0.021 | COG2740: Predicted nucleic-acid-binding protein implicated in transcription termination | gene_0304 |  | RNA Metabolism |
| 139 | ± | 44 | 399 | ± | 19 | -2.08 | * | 0.020 | TPR-repeat-containing protein | gene_0904 |  | ND |
| 45 | ± | 29 | 131 | ± | 16 | -2.11 | * | 0.028 | CRISPR-associated RAMP protein, Csm4 family | gene_0977 |  | DNA Metabolism |
| 194 | ± | 82 | 585 | ± | 88 | -2.12 | * | 0.017 | DinG family ATP-dependent helicase YoaA | gene_0748 |  | DNA Metabolism |
| 89 | ± | 19 | 270 | ± | 80 | -2.16 | * | 0.049 | Transcriptional regulator SpxA2 | gene_0048 |  | DNA Metabolism |
| 21 | ± | 9 | 61 | ± | 12 | -2.18 | * | 0.045 | NADPH-dependent 7-cyano-7-deazaguanine reductase | gene_0762 | 1.7.1.13 | Amino Acids and Derivatives |
| 116 | ± | 18 | 342 | ± | 68 | -2.18 | * | 0.026 | Glutamine ABC transporter, periplasmic glutamine-binding protein (TC 3.A.1.3.2) | gene_0927 |  | Membrane Transport |
| 244 | ± | 158 | 721 | ± | 100 | -2.19 | * | 0.033 | CRISPR-associated protein, Csm1 family | gene_0972 |  | DNA Metabolism |
| 76 | ± | 28 | 244 | ± | 18 | -2.29 | * | 0.012 | Alpha-L-Rha alpha-1,3-L-rhamnosyltransferase | gene_1397 | 2.4.1 | Cell Wall and Capsule |
| 28 | ± | 26 | 98 | ± | 64 | -2.29 | * | 0.046 | hypothetical protein, truncated | gene_0367 |  | ND |
| 89 | ± | 12 | 271 | ± | 92 | -2.31 | * | 0.025 | Flavodoxin | gene_0951 |  | Cofactors, Vitamins, Prosthetic Groups, Pigments |
| 237 | ± | 48 | 751 | ± | 119 | -2.35 | * | 0.034 | Lysyl aminopeptidase | gene_1021 | 3.4.11.15 | Protein Metabolism |
| 69 | ± | 54 | 261 | ± | 175 | -2.37 | * | 0.013 | Mobile element protein | gene_0387 |  | ND |
| 19 | ± | 13 | 70 | ± | 24 | -2.49 | * | 0.011 | FIG01119184: hypothetical protein | gene_1141 |  | ND |
| 7 | ± | 3 | 34 | ± | 18 | -2.72 | * | 0.019 | Twin-arginine translocation protein TatAd | gene_1035 |  | Membrane Transport |
| 171 | ± | 62 | 648 | ± | 358 | -2.81 | * | 0.044 | LSU ribosomal protein L32p | gene_1713 | 3.1.26.12 | Protein Metabolism |
| 246 | ± | 62 | 853 | ± | 377 | -2.81 | * | 0.024 | Urease accessory protein UreF | gene_0152 | 3.1.26.12 | Amino Acids and Derivatives |
| 2 | ± | 3 | 10 | ± | 4 | -2.93 | * | 0.033 | hypothetical protein | gene_0434 |  | ND |
| 7 | ± | 2 | 31 | ± | 13 | -3.22 | * | 0.023 | hypothetical protein | gene_0749 |  | ND |

Table S2 Analysis of genes enrichment in specific functional classes.

Statistically significant category and subcategory of genes differentially expressed. The number of samplings was 5000.

|  | Seed Category and Subcategory | Genes in category | p-val |
| --- | --- | --- | --- |
| Upregulated | Carbohydrates | 14 | 0 |
|  | Di- and oligosaccharides | 5 | 0 |
|  | Monosaccharides | 5 | 0 |
|  | DNA replication | 4 | 0 |
|  | Lysine, threonine, methionine, and cysteine | 4 | 0 |
|  | Capsular and extracellular polysacchrides | 3 | 0 |
|  | Fermentation | 3 | 0 |
|  | Heat shock | 3 | 0 |
|  | Protein degradation | 3 | 0 |
|  | RNA processing and modification | 3 | 0 |
|  | Stress Response | 5 | 0.0198 |
|  |  |  |  |
| Downregulated | Arginine; urea cycle, polyamines | 5 | 0 |
|  | Amino Acids and Derivatives | 12 | 0.0002 |

Table S3 List of predicted genes controlled by CcpA in TH1436 and TH1477 genome.

Genes with higher probability of containing *cre* sequence in their promoter region. Three groups were created according to the presence of genes in the two strains analyzed.

| **Strain** | **Gene** | **Product** | **EC code** | **Level of expression** |
| --- | --- | --- | --- | --- |
| **TH1436 and TH1477** | ackA | Acetate kinase |  | 5.17 |
|  | CcpA | Catabolite control protein A |  | 4.48 |
|  | GlpR | Transcriptional repressor of the fructose operon, DeoR family |  |  |
|  | FruK | 1-phosphofructokinase | 2.7.1.56 | 2.89 |
|  |  | Membrane-bound protease, CAAX family |  |  |
|  | Pgm | Phosphoglucomutase | 5.4.2.2 |  |
|  | AcoA | Acetoin dehydrogenase E1 component alpha-subunit | 1.2.4.- |  |
|  | AcoB | Acetoin dehydrogenase E1 component beta-subunit | 1.2.4.- |  |
|  | AceF | Dihydrolipoamide acetyltransferase component (E2) of acetoin dehydrogenase complex | 2.3.1.- |  |
|  | Lpd | Pyridine nucleotide-disulfide oxidoreductase |  |  |
|  | BglB | 6-phospho-beta-glucosidase | 3.2.1.86 |  |
|  | HprK | HPr kinase/phosphorylase |  |  |
|  | Lgt | Prolipoprotein diacylglyceryltransferase | 2.4.99.- |  |
|  | RsmC | Ribosomal RNA small subunit methyltransferase C | 2.1.1.52 |  |
|  | DeoA | Thymidine phosphorylase | 2.4.2.2 |  |
|  | SunT | ABC-type bacteriocin/lantibiotic exporters, contain an N-terminal double-glycine peptidase domain, Competence-stimulating peptide ABC transporter ATP-binding protein ComA |  |  |
|  | TypA | GTP-binding protein TypA/BipA,membrane GTPase involved in stress response |  |  |
|  | UbiB | Dihydroorotate dehydrogenase electron transfer subunit | 1.3.3.1 |  |
|  | PyrD | Dihydroorotate dehydrogenase, catalytic subunit | 1.3.3.1 |  |
|  |  | Oligoendopeptidase F |  |  |
|  |  | Type II CRISPR RNA-guided endonuclease Cas9 |  |  |
|  |  | Glycogen synthase |  |  |
|  |  | 4-oxalocrotonate tautomerase | 5.3.2.- |  |
|  |  |  |  |  |
| **TH1436** | GalE | UDP-glucose 4-epimerase |  | 4.92 |
|  | GalK | Galactokinase |  | 6.33 |
|  | GalT | Galactose-1-phosphate uridyltransferase |  | 6.43 |
|  | Pta | Phosphate acetyltransferase | 2.3.1.8 | 3.27 |
|  | PtsG | PTS system, sucrose-specific IIA-B-C component | 2.7.1.69 | 8.56 |
|  | - | ACT domain containing transcriptional regulators, related to gcvR of E.coli |  |  |
|  |  | Predicted metal-dependent hydrolase |  |  |
|  |  | Phage transcriptional repressor |  |  |
|  |  | General stress protein, Chemotaxis protein |  |  |
|  | CpsB | Tyrosine-protein phosphatase | 3.1.3.48 |  |
|  | LytR | Exopolysaccharide biosynthesis transcriptional activator EpsA |  |  |
|  | AdhP | Alcohol dehydrogenase | 1.1.1.1 |  |
|  | Asd | Aspartate-semialdehyde dehydrogenase | 1.2.1.11 |  |
|  | DeoB | Phosphopentomutase | 5.4.2.7 |  |
|  | RpiA | Ribose 5-phosphate isomerase |  |  |
|  | DinP | DNA polymerase IV | 2.7.7.7 |  |
|  | Eno | Enolase | 4.2.1.11 |  |
|  | FruB | Phosphotransferase system, HPr-related proteins |  |  |
|  | GloA | Lactoylglutathione lyase |  |  |
|  | SrtA | Sortase A, LPXTG specific |  |  |
|  | GyrA | DNA gyrase subunit A | 5.99.1.3 |  |
|  | HcaD | NADH peroxidase | 1.11.1.1 |  |
|  | NorM | Na+-driven multidrug efflux pump, Multi antimicrobial extrusion (MATE) family transporter |  |  |
|  | ThrC | Threonine synthase | 4.2.3.1 |  |
|  | EutG | Alcohol dehydrogenase, class IV |  |  |
|  | PutA | Alcohol dehydrogenase | 1.1.1.1 |  |
|  | PgsA | Putative enzyme of poly-gamma-glutamate biosynthesis (capsule formation) |  |  |
|  | RplD | LSU ribosomal protein L4p (L1e) |  |  |
|  |  |  |  |  |
| **TH1477** | AcuB | Acetoin utilization acuB protein |  | 3.33 |
|  |  | Fumarate reductase, flavoprotein subunit precursor | 1.3.99.1 | 4.22 |
|  |  | Ribosomal subunit interface protein |  | 3.46 |
|  | PflD | Pyruvate-formate lyase |  | 3.42 |
|  |  | PTS system, mannose-specific IIAB component | 2.7.1.69 | 1.80 |
|  |  | DNA topology modulation protein flar-related protein |  |  |
|  | Ald | Alanine dehydrogenase |  |  |
|  | ArgE | Acetylornithine deacetylase/Succinyl-diaminopimelate desuccinylase and related deacylases |  |  |
|  | CoaA | Panthothenate kinase |  |  |
|  | Dut | Deoxyuridine 5'-triphosphate nucleotidohydrolase | 3.6.1.23 |  |
|  | Fba | Fructose-bisphosphate aldolase class II | 4.1.2.13 |  |
|  | FtsW | Cell division protein |  |  |
|  |  | Pyruvate oxidase |  |  |
|  | LivK | High-affinity leucine-specific transport system periplasmic binding protein LivK |  |  |
|  | LDH | L-lactate dehydrogenase |  |  |
|  | LacS | Lactose permease |  |  |
|  | MutY | A/G-specific adenine glycosylase | 3.2.2.- |  |
|  | Pfk | 6-phosphofructokinase |  |  |
|  | PrfC | Peptide chain release factor RF-3 |  |  |
|  | RplR | LSU ribosomal protein L18p (L5e) |  |  |
|  | TdcF | Endoribonuclease L-PSP |  |  |
|  |  | ACT domain-containing protein transcriptional regulators |  |  |
|  |  | Aminoacylase/Catalyzes the cleavage of p-aminobenzoyl-glutamate to p-aminobenzoate and glutamate subunit A |  |  |
|  |  | PTS system, glucose-specific IIBC component | 2.7.1.69 |  |
