# Supplementary material for: Comparative Transcriptomic Analysis of Streptococcus thermophilus TH1436 and TH1477 Showing Different Capability in the Use of Galactose
Source: Front Microbiol. 2018 Aug 7;9:1765. doi: 10.3389/fmicb.2018.01765 (PMC6090898; doi:10.3389/fmicb.2018.01765)
Supplement: Supplementary file 2 [file Data_Sheet_1.docx]

Fig. S1 Gene alignments of *gal-lac* operon

Complete genes sequences of *galR*, *galK*, *galT*, *galE*, *galM*, *lacS* and *lacZ* of the 6 strains were aligned. Strain LMG18311 was used as reference sequence.
