# Supplementary material for: Comparative Transcriptomic Analysis of Streptococcus thermophilus TH1436 and TH1477 Showing Different Capability in the Use of Galactose
Source: Front Microbiol. 2018 Aug 7;9:1765. doi: 10.3389/fmicb.2018.01765 (PMC6090898; doi:10.3389/fmicb.2018.01765)
Supplement: Supplementary file 5 [file Data_Sheet_4.docx]

Fig. S2 Intergenic region alignment between *galE*-*galM,* *galM*-*lacS*

The intergenic sequences of the 6 strains were aligned. Strain LMG18311 was used as reference sequence.
