# Supplementary material for: Comparative Transcriptomic Analysis of Streptococcus thermophilus TH1436 and TH1477 Showing Different Capability in the Use of Galactose
Source: Front Microbiol. 2018 Aug 7;9:1765. doi: 10.3389/fmicb.2018.01765 (PMC6090898; doi:10.3389/fmicb.2018.01765)
Supplement: Supplementary file 4 [file Data_Sheet_2.docx]

galE

```
LMG18311      ATGGCAATTTTAGTATTAGGTGGAGCTGGTTATATCGGTTCTCACATGGTTGATCGCTTA      60
TH1436        atggcaattttagtattaggtggagctggttatatcggttctcacatggttgatcgctta      60
TH1477        atggcaattttagtattaggtggagctggttatatcggttctcacatggttgatcgctta      60
TH1435        atggcaattttagtattaggtggagctggttatatcggttctcacatggttgatcgctta      60
1F8CT         atggcaattttagtattaggtggagctggttatatcggttctcacatggttgatcgctta      60
TH985         atggcaattttagtattaggtggagctggttatatcggttctcacatggttgatcgctta      60
TH982         atggcaattttagtattaggtggagctggttatatcggttctcacatggttgatcgctta      60
              ************************************************************

LMG18311      GTTGAAAAAGGTCAAGAAAAAGTAGTAGTAGTAGATAGTTTGGTAACAGGTCACCGTGCT      120
TH1436        gttgaaaaaggtcaagaaaaagtagtagtagtagatagtttggtaacaggtcaccgtgct      120
TH1477        gttgaaaaaggtcaagaaaaagtagtagtagtagatagtttggtaacaggtcaccgtgct      120
TH1435        gttgaaaaaggtcaagaaaaagtagtagtagtagatagtttggtaacaggtcaccgtgct      120
1F8CT         gttgaaaaaggtcaagaaaaagtagtagtagtagatagtttggtaacaggtcaccgtgct      120
TH985         gttgaaaaaggtcaagaaaaagtagtagtagtagatagtttggtaacaggtcaccgtgct      120
TH982         gttgaaaaaggtcaagaaaaagtagtagtagtagatagtttggtaacaggtcaccgtgct      120
              ************************************************************

LMG18311      GCCGTTCATCCAGATGCTATTTTTTATCAAGGCGATCTTTCTGATCAGGATTTTATGAGA      180
TH1436        gccgtacatccagatgctattttctatcaaggcgatctttctgatcaggattttatgaga      180
TH1477        gccgtacatccagatgctattttttatcaaggcgatctttctgatcaggattttatgaga      180
TH1435        gccgtacatccagatgctattttttatcaaggcgatctttctgatcaggattttatgaga      180
1F8CT         gccgtacatccagatgctattttctatcaaggcgatctttctgatcaggattttatgaga      180
TH985         gccgtacatccagatgctattttctatcaaggcgatctttctgatcaggattttatgaga      180
TH982         gccgtacatccagatgctattttctatcaaggcgatctttctgatcaagattttatgaga      180
              ***** ***************** *********************** ************

LMG18311      AAGGTCTTCAAAGAAAACCCTGATGTTGATGCTGTTATTCACTTTGCGGCCTATTCTTTG      240
TH1436        aaggtcttcaaagaaaaccctgatgttgatgctgttattcactttgcggcctattctttg      240
TH1477        aaggtcttcaaagaaaaccctgatgttgatgctgttattcactttgcggcctattctttg      240
TH1435        aaggtcttcaaagaaaaccctgatgttgatgctgttattcactttgcggcctattctttg      240
1F8CT         aaggtcttcaaagaaaaccctgatgttgatgctgttattcactttgcggcctattctttg      240
TH985         aaggtcttcaaagaaaaccctgatgttgatgctgttattcactttgcggcctattctttg      240
TH982         aaggtcttcaaagaaaaccctgatgttgatgctgttattcactttgcggcctattctttg      240
              ************************************************************

LMG18311      GTTGGTGAATCAATGGAAAAACCACTCAAATATTTTGATAACAACACAGCTGGAATGATT      300
TH1436        gttggtgaatcaatggaaaaaccactcaaatattttgataacaacacagctggaatggtt      300
TH1477        gttggtgaatcaatggaaaaaccactcaaatattttgataacaacacagctggaatggtt      300
TH1435        gttggtgaatcaatggaaaaaccactcaaatattttgataacaacacagctggaatggtt      300
1F8CT         gttggtgaatcaatggaaaaaccactcaaatattttgataacaacacagctggaatggtt      300
TH985         gttggtgaatcaatggaaaaaccactcaaatattttgataacaacacagctggaatggtt      300
TH982         gttggtgaatcaatggaaaaaccactcaaatattttgataacaacacagctggaatggtt      300
              ********************************************************* **

LMG18311      AAATTGCTTGAGGTTATGAACGAATGCGGTGTTAAATACATTGTCTTCTCATCAACAGCA      360
TH1436        aaattgcttgaggttatgaacgaatgcggtgttaaatacattgtcttctcatcaacagca      360
TH1477        aaattgcttgaggttatgaacgaatgtggtgttaaatacattgtcttctcatcaacagca      360
TH1435        aaattgcttgaggttatgaacgaatgtggtgttaaatacattgtcttctcatcaacagca      360
1F8CT         aaattgcttgaggttatgaacgaatgcggtgttaaatacattgtcttctcatcaacagca      360
TH985         aaattgcttgaggttatgaacgaatgcggtgttaaatacattgtcttctcatcaacagca      360
TH982         aaattgcttgaggttatgaacgaatgcggtgttaaatacattgtcttctcatcaacagca      360
              ************************** *********************************

LMG18311      GCAACTTACGGAATTCCAGAAGAAATTCCAATTCTTGAAACAACACCACAAAATCCTATC      420
TH1436        gcaacttacggaattccagaagaaattccaattcttgaaacaacaccacaaaatcctatc      420
TH1477        gcaacttacggaattccagaagaaattccaattcttgaaacaacaccacaaaatcctatc      420
TH1435        gcaacttacggaattccagaagaaattccaattcttgaaacaacaccacaaaatcctatc      420
1F8CT         gcaacttacggaattccagaagaaattccaattcttgaaacaacaccacaaaatcctatc      420
TH985         gcaacttacggaattccagaagaaattccaattcttgaaacaacaccacaaaatcctatc      420
TH982         gcaacttacggaattccagaagaaattccaattcttgaaacaacaccacaaaatcctatc      420
              ************************************************************

LMG18311      AACCCATATGGTGAAAGTAAGTTGATGATGGAAACCATTATGAAGTGGTCAGACCAAGCT      480
TH1436        aacccatatggtgaaagtaagttgatgatggaaactattatgaggtggtcagaccaagtt      480
TH1477        aacccatatggtgaaagtaagttgatgatggaaaccattatgaagtggtcagaccaagct      480
TH1435        aacccatatggtgaaagtaagttgatgatggaaaccattatgaagtggtcagaccaagct      480
1F8CT         aacccatatggtgaaagtaagttgatgatggaaaccattatgaagtggtcagaccaagct      480
TH985         aacccatatggtgaaagtaagttgatgatggaaaccattatgaagtggtcagaccaagct      480
TH982         aacccatatggtgaaagtaagttgatgatggaaaccattatgaagtggtcagaccaagct      480
              *********************************** ******* ************** *
```

```
LMG18311      TACGGCATCAAGTATGTCCCTCTTCGTTACTTTAATGTGGCGGGTGCCAAACCTGATGGT      540
TH1436        tacggcatcaagtatgtccctcttcgttacttcaacgttgctggtgccaaacctgatggt      540
TH1477        tacggcatcaagtatgtccctcttcgttactttaatgtggcgggtgccaaacctgatggt      540
TH1435        tacggcatcaagtatgtccctcttcgttactttaatgtggcgggtgccaaacctgatggt      540
1F8CT         tacggcatcaagtatgtccctcttcgttactttaatgtggcgggtgccaaacctgatggt      540
TH985         tacggcatcaagtatgtccctcttcgttactttaatgtggcgggtgccaaacctgatggt      540
TH982         tacggcatcaagtatgtccctcttcgttactttaatgtggcgggtgccaaacctgatggt      540
              ******************************** ** ** ** ******************

LMG18311      TCGATTGGTGAGGACCACGATCCAGAAACTCACCTTCTACCGATTATTCTTCAAGTAGCT      600
TH1436        tcgattggtgaggaccacggtccagaaactcaccttctaccgattattcttcaagtagct      600
TH1477        tcgattggtgaggaccacggtccagaaactcaccttctaccgattattcttcaagtagct      600
TH1435        tcgattggtgaggaccacggtccagaaactcaccttctaccgattattcttcaagtagct      600
1F8CT         tcgattggtgaggaccacgatccagaaactcaccttctaccgattattcttcaagtagct      600
TH985         tcgattggtgaggaccacgatccagaaactcaccttctaccgattattcttcaagtagct      600
TH982         tcgattggtgaggaccacgatccagaaactcaccttctaccgattattcttcaagtagct      600
              ******************* ****************************************

LMG18311      CAAGGTGTTCGTGAAAAAATCATGATCTTTGGGGATGACTACAATACTCCAGACGGGACT      660
TH1436        caaggtgttcgtgaaaaaatcatgatctttggggatgactacaatactccagacgggact      660
TH1477        caaggtgttcgtgaaaaaatcatgatctttggggatgactacaatactccagacgggact      660
TH1435        caaggtgttcgtgaaaaaatcatgatctttggggatgactacaatactccagacgggact      660
1F8CT         caaggtgttcgtgaaaaaatcatgatctttggggatgactacaatactccagacgggact      660
TH985         caaggtgttcgtgaaaaaatcatgatctttggggatgactacaatactccagacgggact      660
TH982         caaggtgttcgtgaaaaaatcatgatctttggggatgactacaatactccagacgggact      660
              ************************************************************

LMG18311      AACGTTCGTGACTATGTGCATCCATTCGACTTGGCGGACGCTCACCTCCTTACTGTTGAA      720
TH1436        aacgttcgtgactatgtgcatccattcgacttggcggacgctcacctccttgctgttgaa      720
TH1477        aacgttcgtgactatgtgcatccattcgacttggcggacgctcacctccttgctgttgaa      720
TH1435        aacgttcgtgactatgtgcatccattcgacttggcggacgctcacctccttgctgttgaa      720
1F8CT         aacgttcgtgactatgtgcatccattcgacttggcggacgctcacctccttgctgttgaa      720
TH985         aacgttcgtgactatgtgcatccattcgacttggcggacgctcacctccttgctgttgaa      720
TH982         aacgttcgtgactatgtgcatccattcgacttggcggacgctcacctccttgctgttgaa      720
              *************************************************** ********

LMG18311      TATCTTCGTAAAGGTAATGAATCTACAGCCTTAGACTTAGGTTCATCAACAGGTTTCTCA      780
TH1436        tatcttcgtaaaggtaatgaatctacagcctttaacttaggttcatcaacaggtttctca      780
TH1477        tatcttcgtaaaggtaatgaatctacagcctttaacttaggttcatcaacaggtttctca      780
TH1435        tatcttcgtaaaggtaatgaatctacagcctttaacttaggttcatcaacaggtttctca      780
1F8CT         tatcttcgtaaaggtaatgaatctacagcctttaacttaggttcatcaacaggtttctca      780
TH985         tatcttcgtaaaggtaatgaatctacagcctttaacttaggttcatcaacaggtttctca      780
TH982         tatcttcgtaaaggtaatgaatctacagcctttaacttaggttcatcaacaggtttctca      780
              ********************************  **************************

LMG18311      AACCTTCAAATTCTTGAAGCAGCTTGTAAGGTAACTGGTAAAGAGATTCCAGCTGAAAAA      840
TH1436        aaccttcaaattcttgaagcagctcgtaaggtaactggtaaagagattccagctgaaaaa      840
TH1477        aaccttcaaattcttgaagcagctcgtaaggtaactggtaaagagattccagctgaaaaa      840
TH1435        aaccttcaaattcttgaagcagctcgtaaggtaactggtaaagagattccagctgaaaaa      840
1F8CT         aaccttcaaattcttgaagcagctcgtaaggtaactggtaaagagattccagctgaaaaa      840
TH985         aaccttcaaattcttgaagcagctcgtaaggtaactggtaaagagattccagctgaaaaa      840
TH982         aaccttcaaattcttgaagcagctcgtaaggtaactggtaaagagattccagctgaaaaa      840
              ************************ ***********************************

LMG18311      GCTGATCGTCTTCGTGGTGATCCTGATATATTGATTGCTTCATCTGAAAAAGCACGTACA      900
TH1436        gctgatcgtcgtcctggtgatcctgatatattgattgcttcatctgaaaaagcacgtaca      900
TH1477        gctgatcgtcgtcctggtgatcctgatatattgattgcttcatctgaaaaagcacgtaca      900
TH1435        gctgatcgtcgtcctggtgatcctgatatattgattgcttcatctgaaaaagcacgtaca      900
1F8CT         gctgatcgtcgtcctggtgatcctgatatattgattgcttcatctgaaaaagcacgtaca      900
TH985         gctgatcgtcgtcctggtgatcctgatatattgattgcttcatctgaaaaagcacgtaca      900
TH982         gctgatcgtcgtcctggtgatcctgatatattgattgcttcatctgaaaaagcacgtaca      900
              ********** ** **********************************************

LMG18311      GTTTTTGGATGGAAACCACAATTTGATAACATCGACAATATCATCGCTAGTGCTTGGGCA      960
TH1436        gttcttggatggaaaccacaatttgataacatcgaaaaaatcatcgcaagtgcttgggca      960
TH1477        gttcttggatggaaaccacaatttgataacatcgaaaaaatcatcgcaagtgcttgggca      960
TH1435        gttcttggatggaaaccacaatttgataacatcgaaaaaatcatcgcaagtgcttgggca      960
1F8CT         gttcttggatggaaaccacaatttgataacatcgaaaaaatcatcgcaagtgcttgggca      960
TH985         gttcttggatggaaaccacaatttgataacatcgaaaaaatcatcgcaagtgcttgggca      960
TH982         gttcttggatggaaaccacaatttgataacatcgaaaaaatcatcgcaagtgcttgggca      960
              *** ******************************* ** ******** ************

LMG18311      TGGCATTCTAACCATCCTAAAGGGTACGATGATCGAGGATTATTAAAATAA      1011
TH1436        tggcattctagccatccaaaagggtacgatgatcgaggataa---------      1002
TH1477        tggcattctagccatccaaaagggtacgatgatcgaggataa---------      1002
TH1435        tggcattctagccatccaaaagggtacgatgatcgaggataa---------      1002
1F8CT         tggcattctagccatccaaaagggtacgatgatcgaggataa---------      1002
TH985         tggcattctagccatccaaaagggtacgatgatcgaggataa---------      1002
TH982         tggcattctagccatccaaaagggtacgatgatcgaggataa---------      1002
              ********** ****** ********************** *
```

galM

```
TH1436      atgaaaataagctgtgaaattattggaaaagttgattcaggcgatgtcagtaaaatttca    60
TH1435      atgaaaataagctgcgaaattattggaaaagttgattcaggcgatgtcagtaaaatttca    60
TH982       atgaaaataagctgcgaaattattggaaaagttgattcaggcgatgtcagtaaaatttca    60
LMG18311    ATGAAAATAAGCTGCGAAATTATTGGAAAAGTTGATTCAGGCGATGTCAGTAAAATTTCA    60
TH1477      atgaaaataagctgcgaaattattggaaaagttgattcaggcgatgtcagtaaaatttca    60
1F8CT       atgaaaataagctgcgaaattattggaaaagttgattcaggcgatgtcagtaaaatttca    60
TH985       atgaaaataagctgcgaaattattggaaaagttgattcaggcgatgtcagtaaaatttca    60
            ************** *********************************************

TH1436      atggaaaacaataatggtgttgtcatttccacactcacaacaggtgctacacttcaggag    120
TH1435      atggaaaataataatggtgttgtcatttccacactcacaacaggtgctacacttcaggag    120
TH982       atggaaaataataatggtgttgtcatttccacactcacaacaggtgctacacttcaggag    120
LMG18311    ATGGAAAATAATAATGGTGTTGTCATTTCCACACTCACAACAGGTGCTACACTTCAGGAG    120
TH1477      atggaaaataataatggtgttgtcatttccacactcacaacaggtgctacacttcaggag    120
1F8CT       atggaaaataataatggtgttgtcatttccacactcacaacaggtgctacacttcaggag    120
TH985       atggaaaataataatggtgttgtcatttccacactcacaacaggtgctacacttcaggag    120
            ******** ***************************************************

TH1436      tttttggttccaacggaaactggtgctcttaaaaatatagtacttggtttcagtgatttc    180
TH1435      tttttggttccaatggaaactggtgctcttaaaaatatagtacttggtttcagtgatttc    180
TH982       tttttggttccaacggaaactggtgctcttaaaaatatagtacttggtttcagtgatttc    180
LMG18311    TTTTTGGTTCCAACGGAAACTGGTGCTCTTAAAAATATAGTACTTGGTTTCAGTGATTTC    180
TH1477      tttttggttccaacggaaactggtgctcttaaaaatatagtacttggtttcagtgatttc    180
1F8CT       tttttggttccaacggaaactggtgctcttaaaaatatagtacttggtttcagtgatttc    180
TH985       tttttggttccaacggaaactggtgctcttaaaaatatagtacttggtttcagtgatttc    180
            ************* **********************************************

TH1436      gaggattactataagaataatttatgtgcctgccagtccattggtagggttgctggaaga    240
TH1435      gaggattactataagaataatttatgtgcctgccagtccattggtagggttgctggaaga    240
TH982       gaggattactataagaataatttatgtgcctgccagtccattggtagggttgctggaaga    240
LMG18311    GAGGATTACTATAAGAATAATTTATGTGCCTGCCAGTCCATTGGTAGGGTTGCTGGAAGA    240
TH1477      gaggattactataagaataatttatgtgcctgccagtccattggtagggttgctggaaga    240
1F8CT       gaggattactataagaataatttatgtgcctgccagtccattggtagggttgctggaaga    240
TH985       gaggattactataagaataatttatgtgcctgccagtccattggtagggttgctggaaga    240
            ************************************************************

TH1436      attgggaaagcttcgtatactcataatatggttctttatagccttcctaagaatgaggga    300
TH1435      attgggaaagcttcgtatactcataatatggttctttatagccttcctaagaatgaggga    300
TH982       attgggaaagcttcgtatactcataatatggttctttatagccttcctaagaatgaggga    300
LMG18311    ATTGGGAAAGCTTCGTATACTCATAATATGGTTCTTTATAGCCTTCCTAAGAATGAGGGA    300
TH1477      attgggaaagcttcgtatactcataatatggttctttatagccttcctaagaatgaggga    300
1F8CT       attgggaaagcttcgtatactcataatatggttctttatagccttcctaagaatgaggga    300
TH985       attgggaaagcttcgtatactcataatatggttctttatagccttcctaagaatgaggga    300
            ************************************************************

TH1436      gaaaactgcttacatggcggtccaaaaggaatgcaggttcaaaattggaactatgtcaca    360
TH1435      gacaactgcttacatggcggtccaaaaggaatgcaggttcaaaattggaactatgtcaca    360
TH982       gaaaactgcttacatggcggtccaaaaggagtgcaggtccaaaattggaactatgtcaca    360
LMG18311    GAAAACTGCTTACATGGCGGTCCAAAAGGAATGCAGGTCCAAAATTGGAACTATGTCACA    360
TH1477      gaaaactgcttacatggcggtccaaaaggaatgcaggtccaaaattggaactatgtcaca    360
1F8CT       gaaaactgcttacatggcggtccaaaaggaatgcaggtccaaaattggaactatgtcaca    360
TH985       gaaaactgcttacatggcggtccaaaaggaatgcaggtccaaaattggaactatgtcaca    360
            ** *************************** ******* *********************

TH1436      aacctgaatgatgaatatgttgagacaaaatttattagaagactttattcaagtgtagat    420
TH1435      aacctgaatgatgattatgttgagacaaaatttattagaagactttattcaagtgtagat    420
TH982       aacctgaatgatgaatatgttgagacaaaatttattagaagactttattcaagtgtagat    420
LMG18311    AACCTGAATGATGAATATGTTGAGACAAAATTTATTAGAAGACTTTATTCAAGTGTAGAT    420
TH1477      aacctgaatgatgaatatgttgagacaaaatttattagaagactttattcaagtgtagat    420
1F8CT       aacctgaatgatgaatatgttgagacaaaatttattagaagactttattcaagtgtagat    420
TH985       aacctgaatgatgaatatgttgagacaaaatttattagaagactttattcaagtgtagat    420
            ************** *********************************************

TH1436      ggctttcctggtgatgtgacagtctctattagttatagacttaacaataataaccgctta    480
TH1435      ggatttcctggtgatgtgacagtctctattagttatagacttaacaataataaccgctta    480
TH982       ggctttcctggtgatgtgacagtctctattagttatagacttaacaataataaccgctta    480
LMG18311    GGCTTTCCTGGTGATGTGACAGTCTCTATTAGTTATAGACTTAACAATAATAACCGCTTA    480
TH1477      ggctttcctggtgatgtgacagtctctattagttatagacttaacaataataaccgctta    480
1F8CT       ggctttcctggtgatgtgacagtctctattagttatagacttaacaataataaccgctta    480
TH985       ggctttcctggtgatgtgacagtctctattagttatagacttaacaataataaccgctta    480
            ** *********************************************************
```

```
TH1436      acaatactctttgaagccttcgatgttacagagtcatcagtctttaatccgacaaaccat    540
TH1435      acaatactctttgaagccttcgatgttacagagtcaacagtctttaatccgacaaaccat    540
TH982       acaatactctttgaagccttcgatgttacagagtcaacaattttaatccgacaaaccat    540
LMG18311    ACAATACTCTTTGAAGCCTTCGATGTTACAGAGTCAACAATTTTAATCCGACAAACCAT    540
TH1477      acaatactctttgaagccttcgatgttacagagtcaacaattttaatccgacaaaccat    540
1F8CT       acaatactctttgaagccttcgatgttacagagtcaacaattttaatccgacaaaccat    540
TH985       acaatactctttgaagccttcgatgttacagagtcaacaattttaatccgacaaaccat    540
            ************************************ ** * ******************

TH1436      gtttactttaatcttagcgacaaacaggatttatcaagtcatgagttacaaatctattca    600
TH1435      gtttactttaatcttagcgacaaacaggatttatcaagtcatgagttacaaatctattca    600
TH982       gtttactttaatcttagcgacaaacaggatttatcaagtcatgagttacaaatctattca    600
LMG18311    GTTTACTTTAATCTTAGCGACAAACAGGATTTATCAAGTCATGAGTTACAAATCTATTCA    600
TH1477      gtttactttaatcttagcgacaaacaggatttatcaagtcatgagttacaaatctattca    600
1F8CT       gtttactttaatcttagcgacaaacaggatttatcaagtcatgagttacaaatctattca    600
TH985       gtttactttaatcttagcgacaaacaggatttatcaagtcatgagttacaaatctattca    600
            ************************************************************

TH1436      gactatcgtttagagttggattctgagttaattccaacaggacaaaaaattaatgtagat    660
TH1435      gactatcgtttagagttggattctgagttaattccaacaggacaaaaaattaatgtagat    660
TH982       gactatcgtttagagttggattctgagttaattccaacaggacaaaaaattaatgtagat    660
LMG18311    GACTATCGTTTAGAGTTGGATTCTGAGTTAATTCCAACAGGACAAAAAATTAATGTAGAT    660
TH1477      gactatcgtttagagttggattctgagttaattccaacaggacaaaaaattaatgtagat    660
1F8CT       gactatcgtttagagttggattctgagttaattccaacaggacaaaaaattaatgtagat    660
TH985       gactatcgtttagagttggattctgagttaattccaacaggacaaaaaattaatgtagat    660
            ************************************************************

TH1436      gaaacgaattatgattttagaaagacaactgacttgctacctcgaattgaagcaaataat    720
TH1435      gaaacgaattatgattttagaaagacaactgacttgctacctcgaattgaagcaaataat    720
TH982       gaaacgaattatgattttagaaagacaactgacttgctacctcgaattgaagcaaataat    720
LMG18311    GAAACGAATTATGATTTTAGAAAGACAACTGACTTGCTACCTCGAATTGAAGCAAATAAT    720
TH1477      gaaacgaattatgattttagaaagacaactgacttgctacctcgaattgaagcaaataat    720
1F8CT       gaaacgaattatgattttagaaagacaactgacttgctacctcgaattgaagcaaataat    720
TH985       gaaacgaattatgattttagaaagacaactgacttgctacctcgaattgaagcaaataat    720
            ************************************************************

TH1436      ggttttgatgatgcctttgtagttggaggggaacttgtgatcatgtgaaagaagtagct    780
TH1435      ggttttgatgatgcctttgtagttggaggggaacttgtgatcatgtgaaagaagtagct    780
TH982       ggttttgatgatgcctttgtagttggaggggaacttgtgatcatgtgaaagaagtagct    780
LMG18311    GGTTTTGATGATGCCTTTGTAGTTGGAGGGGAACTTGTGATCATGTGAAAGAAGTAGCT    780
TH1477      ggttttgatgatgcctttgtagttggaggggaacttgtgatcatgtgaaagaagtagct    780
1F8CT       ggttttgatgatgcctttgtagttggaggggaacttgtgatcatgtgaaagaagtagct    780
TH985       ggttttgatgatgcctttgtagttggaggggaacttgtgatcatgtgaaagaagtagct    780
            ************************************************************

TH1436      atttacatgacaaagaaagcggagatggtattgaaatcttctcaaacagaaatggttta    840
TH1435      atttgcatgacaaagaaagtggagatggtattgaaatcttctcaaacagaaatggttta    840
TH982       atttgcatgacaaagaaagtggagatggtattgaaatcttctcaaacagaaatagttta    840
LMG18311    ATTTGCATGACAAAGAAAGTGGAGATGGTATTGAAATCTTCTCAAACAGAAATGGTTTA    840
TH1477      atttgcatgacaaagaaagtggagatggtattgaaatcttctcaaacagaaatggttta    840
1F8CT       atttgcatgacaaagaaagtggagatggtattgaaatcttctcaaacagaaatagttta    840
TH985       atttgcatgacaaagaaagtggagatggtattgaaatcttctcaaacagaaatagttta    840
            ***** ************** ********************************* *****

TH1436      gtcatttttacgatggatgatatcgaagataatatttactttgcaagagataaaggcaaa    900
TH1435      gtcatttttacgatggatgatatcgaagataatatttactttgcaagagataaaggcaaa    900
TH982       gtcatttttacgatggatgatatcgaagataatatttactttgcaagagataaaggcaaa    900
LMG18311    GTCATTTTTACGATGGATGATATCGAAGATAATATTTACTTTGCAAGAGATAAAGGCAAA    900
TH1477      gtcatttttacgatggatgatatcgaagataatatttactttgcaagagataaaggcaaa    900
1F8CT       gtcatttttacgatggatgatatcgaagataatatttactttgcaagagataaaggcaaa    900
TH985       gtcatttttacgatggatgatatcgaagataatatttactttgcaagagataaaggcaaa    900
            ************************************************************

TH1436      atggcaaaagggcgtgaagctattgctatggaagctcaaacccttccagatgccattaat    960
TH1435      atggcaaaaaggcgtgaagctattgctatggaagctcaaacccttccagatgccgttaat    960
TH982       atggcaaaaaggcgtgaagctattgctatggaagctcaaacccttccagatgccgttaat    960
LMG18311    ATGGCAAAAAGGCGTGAAGCTATTGCTATGGAAGCTCAAACCCTTCCAGATGCCGTTAAT    960
TH1477      atggcaaaaaggcgtgaagctattgctatggaagctcaaacccttccagatgccgttaat    960
1F8CT       atggcaaaaaggcgtgaagctattgctatggaagctcaaacccttccagatgccgttaat    960
TH985       atggcaaaaaggcgtgaagctattgctatggaagctcaaacccttccagatgccgttaat    960
            ********* ******************************************** *****

TH1436      cacaaaggttttggagatattatcttagataaaggtcatagtgtaaactatgaaattggc    1020
TH1435      cacaaaggttttggagatattatcttagataaaggtcatagtgtaaactatgaaattggc    1020
TH982       cacaaaggttttggagatattatcttagataaaggtcatagtgtaaactatgaaattggc    1020
LMG18311    CACAAAGGTTTTGGAGATATTATCTTAGATAAAGGTCATAGTGTAAACTATGAAATTGGC    1020
TH1477      cacaaaggttttggagatattatcttagataaaggtcatagtgtaaactatgaaattggc    1020
1F8CT       cacaaaggttttggagatattatcttagataaaggtcatagtgtaaactatgaaattggc    1020
TH985       cacaaaggttttggagatattatcttagataaaggtcatagtgtaaactatgaaattggc    1020
            ************************************************************

TH1436      ttccaatactttaattcatcaagataa    1047
TH1435      ttccaatactttaattcatcaagataa    1047
TH982       ttccaatactttaattcatcaagataa    1047
LMG18311    TTCCAATACTTTAATTCATCAAGATAA    1047
TH1477      ttccaatactttaattcatcaagataa    1047
1F8CT       ttccaatactttaattcatcaagataa    1047
TH985       ttccaatactttaattcatcaagataa    1047
            ***************************
```
