# Supplementary material for: Comparative Transcriptomic Analysis of Streptococcus thermophilus TH1436 and TH1477 Showing Different Capability in the Use of Galactose
Source: Front Microbiol. 2018 Aug 7;9:1765. doi: 10.3389/fmicb.2018.01765 (PMC6090898; doi:10.3389/fmicb.2018.01765)
Supplement: Supplementary file 5 [file Data_Sheet_3.docx]

lacS

```
TH1477      atggaaaaatctaaaggtcagatgaagtctcgtttatcctacgcagctggtgcttttggt   60
TH1435      atggaaaaatctaaaggtcagatgaagtctcgtttatcctacgcagctggtgcttttggt   60
LMG18311    ATGGAAAAATCTAAAGGTCAGATGAAGTCTCGTTTATCCTACGCAGCTGGTGCTTTTGGT   60
TH1436      atggaaaaatctaaaggtcagatgaagtctcgtttatcctacgcagctggtgcttttggt   60
TH985       atggaaaaatctaaaggtcagatgaagtctcgtttatcctacgcagctggtgcttttggt   60
1F8CT       atggaaaaatctaaaggtcagatgaagtctcgtttatcctacgcagctggtgcttttggt   60
TH982       atggaaaaatctaaaggtcagatgaagtctcgtttatcctacgcagctggtgcttttggt   60
            ************************************************************

TH1477      aacgacgtcttctatgcaaccttgtcaacatactttatcatgtttgtgacaactcacttg   120
TH1435      aacgacgtcttctatgcaaccttgtcaacatactttatcatgtttgtgacaactcacttg   120
LMG18311    AACGACGTCTTCTATGCAACCTTGTCAACATACTTTATCATGTTTGTGACAACTCACTTG   120
TH1436      aacgacgtcttctatgcaaccttgtcaacatactttatcatgtttgtgacaactcacttg   120
TH985       aacgacgtcttctatgcaaccttgtcaacatactttatcatgtttgtgacaactcacttg   120
1F8CT       aacgacgtcttctatgcaaccttgtcaacatactttatcatgtttgtgacaactcacttg   120
TH982       aacgacgtcttctatgcaaccttgtcaacatactttatcatgtttgtgacaactcacttg   120
            ************************************************************

TH1477      tttaacacaggtgatccaaagcaaaatagtcactacgtactattaatcactaacattatc   180
TH1435      tttaacacaggtgatccaaagcaaaatagtcactacgtactattaatcactaacattatc   180
LMG18311    TTTAACACAGGTGATCCAAAGCAAAATAGTCACTACGTACTATTAATCACTAACATTATC   180
TH1436      tttaacacaggtgatccaaagcaaaatagtcactacgtactattaatcactaacattatc   180
TH985       tttaacacaggtgatccaaagcaaaatagtcactacgtactattaatcactaacattatc   180
1F8CT       tttaacacaggtgatccaaagcaaaatagtcactacgtactattaatcactaacattatc   180
TH982       tttaacacaggtgatccaaagcaaaatagtcactacgtactattaatcactaacattatc   180
            ************************************************************

TH1477      tctattttgcgtatcttggaagtatttatcgatccattgatcggtaatatgattgataac   240
TH1435      tctattttgcgtatcttggaagtatttatcgatccattgatcggtaatatgattgataac   240
LMG18311    TCTATTTTGCGTATCTTGGAAGTATTTATCGATCCATTGATCGGTAATATGATTGATAAC   240
TH1436      tctattttgcgtatcttggaagtatttatcgatccattgatcggtaatatgattgataac   240
TH985       tctattttgcgtatcttggaagtatttatcgatccattgatcggtaatatgattgataac   240
1F8CT       tctattttgcgtatcttggaagtatttatcgatccattgatcggtaatatgattgataac   240
TH982       tctattttgcgtatcttggaagtatttatcgatccattgatcggtaatatgattgataac   240
            ************************************************************

TH1477      actaatactaagtatggtaaattcaaaccatgggtagttggtggtggtatcatcagttct   300
TH1435      actaatactaagtatggtaaattcaaaccatgggtagttggtggtggtatcatcagttct   300
LMG18311    ACTAATACTAAGTATGGTAAATTCAAACCATGGGTAGTTGGTGGTGGTATCATCAGTTCT   300
TH1436      actaatactaagtatggtaaattcaaaccatgggtagttggtggtggtatcatcagttct   300
TH985       actaatactaagtatggtaaattcaaaccatgggtagttggtggtggtatcatcagttct   300
1F8CT       actaatactaagtatggtaaattcaaaccatgggtagttggtggtggtatcatcagttct   300
TH982       actaatactaagtatggtaaattcaaaccatgggtagttggtggtggtatcatcagttct   300
            ************************************************************

TH1477      atcaccttgttgcttctcttcaccgatttaggtggtttgaataaaacaaatcctttcttg   360
TH1435      atcaccttgttgcttctcttcaccgatttaggtggtttgaataaaacaaatcctttcttg   360
LMG18311    ATCACCTTGTTGTTTCTCTTAACCGATATAGGTGGTTTGAATAAAACAAATCCTTTCTTG   360
TH1436      atcaccttgttgcttctcttcaccgatttaggtggtttgaataaaacaaatcctttcttg   360
TH985       atcaccttgttgcttctcttcaccgatttaggtggtttgaataaaacaaatcctttcttg   360
1F8CT       atcaccttgttgcttctcttcaccgatttaggtggtttgaataaaacaaatcctttcttg   360
TH982       atcaccttgttgcttctcttcaccgatttaggtggtttgaataaaacaaatcctttcttg   360
            ************ ******* ****** ********************************

TH1477      tatcttgtactttttggaattatctaccttgtaatggatgtcttctactcgattaaagat   420
TH1435      taccttgtactttttggaattatctaccttataatggatgtcttctactcgattaaagat   420
LMG18311    TATCTTGTACTTTTTGGAATTATCTACCTTGTAATGGATGTCTTCTACTCGATTAAAGAT   420
TH1436      taccttgtactttttggaattatctaccttgtaatggatgttttctactcgattaaagat   420
TH985       taccttgtactttttggaattatctaccttgtaatggatgtcttctactcgattaaagat   420
1F8CT       taccttgtactttttggaattatctaccttgtaatggatgtcttctactcgattaaagat   420
TH982       taccttgtactttttggaattatctaccttgtaatggatgtcttctactcgattaaagat   420
            ** *************************** ********** ******************

TH1477      atcggtttctggtcaatgattcctgccttgtctcttgatagtcacgaacgtgaaaaaatg   480
TH1435      atcggtttctggtcaatgcttcctgccttgtctcttgacagtcacgaacgtgaaaaaatg   480
LMG18311    ATCGGTTTCTGGTCAATGATTCCTGCCTTGTCTCTTGATAGTCACGAACGTGAAAAAATG   480
TH1436      atcggtttctggtcaatgattcctgccttgtctcttgatagtcacgaacgtgaaaaaatg   480
TH985       atcggtttctggtcaatgattcctgccttgtctcttgatagtcacgaacgtgaaaaaatg   480
1F8CT       atcggtttctggtcaatgattcctgccttgtctcttgatagtcacgaacgtgaaaaaatg   480
TH982       atcggtttctggtcaatgattcctgccttgtctcttgatagtcacgaacgtgaaaaaatg   480
            ****************** ******************* *********************
```

```
TH1477      gcaacttttgcccgtattggttctacgattggtgccaatattgtaggtgttgccatcatg    540
TH1435      gcaacttttgcccgtattggttctacgattggtgccaatattgtaggtgttgccatcatg    540
LMG18311    GCAACTTTTGCCCGTATTGGTTCGACGATTGGTGCCAATATTGTAGGTGTTGCCATCATG    540
TH1436      gcaacttttgcccgtattggttctacgattggtgccaatattgtaggtgttgccatcatg    540
TH985       gcaacttttgcccgtattggttctacgattggtgccaatattgtaggtgttgccatcatg    540
1F8CT       gcaacttttgcccgtattggttctacgattggtgccaatattgtaggtgttgccatcatg    540
TH982       gcaacttttgcccgtattggttctacgattggtgccaatattgtaggtgttgccatcatg    540
            *********************** ************************************

TH1477      ccaatcgttttgttcttctctatgacgaacaatagtggctctggagataaatctggatgg    600
TH1435      ccaatcgttttgttcttctctatgacgaacagtagtggctctggagataaatctggatgg    600
LMG18311    CCAATCGATTTGTTATTCTCTATGACGCACAATAGTGGCTCTGGAGATAAATCTGGATGG    600
TH1436      ccaatcgttttgttcttctctatgacgaacaatagtggctctggagataaatctggatgg    600
TH985       ccaatcgttttgttcttctctatgacgaacaatagtggctctggagataaatctggatgg    600
1F8CT       ccaatcgttttgttcttctctatgacgaacaatagtggctctggagataaatctggatgg    600
TH982       ccaatcgttttgttcttctctatgacgaacaatagtggctctggagataaatctggatgg    600
            ******* ****** ************ *** ****************************

TH1477      ttctggtttgcatttatcgttgctctcattggtgtgattacatcaattgctgttggtatt    660
TH1435      ttctggtttgcatttatcgttgcactcattggtgtgattacatcaattgctgttggtatt    660
LMG18311    TTCTGGTATGCATTTATCGTAGATCTCAATGGTGTGATTACATCAATTGCTGTTGGTATT    660
TH1436      ttctggtttgcatttatcgttgctctcattggtgtgattacatcaattgctgttggtatt    660
TH985       ttctggtttgcatttatcgttgctctcattggtgtgattacatcaattgctgttggtatt    660
1F8CT       ttctggtttgcatttatcgttgctctcattggtgtgattacatcaattgctgttggtatt    660
TH982       ttctggtttgcatttatcgttgctctcattggtgtgattacatcaattgctgttggtatt    660
            ******* ************ *  **** *******************************

TH1477      ggtacacgtgaagttgagtcaaaacttcgtgataacaatgaaaaaactagccttaaacaa    720
TH1435      ggtacacgtgaagttgagtcaaaacttcgtgataacaatgaaaaaactagccttaaacaa    720
LMG18311    GGTACACGTGAAGTTGAGTCAAAAATTCATGATAATAACGAAAAAACTAGCCTTAAACAA    720
TH1436      ggtacacgtgaagttgagtcaaaaattcgtgataataacgaaaaaactagccttaaacaa    720
TH985       ggtacacgtgaagttgagtcaaaaattcgtgataataacgaaaaaactagccttaaacaa    720
1F8CT       ggtacacgtgaagttgagtcaaaaattcgtgataataacgaaaaaactagccttaaacaa    720
TH982       ggtacacgtgaagttgagtcaaaaattcgtgataataacgaaaaaactagccttaaacaa    720
            ************************ *** ****** ** *********************

TH1477      gtctttaaagttcttggtaaaaacgaccaattgatgtggctgtctctcggatattggttc    780
TH1435      gtctttaaagttcttggtaaaaacgaccaattgatgtggctgtctctcggatattggttc    780
LMG18311    GTCTTTAAGGTTCTTGGTCAAAACGACCAAATGATGTGGTTATCTCTTGGATATTGGTTC    780
TH1436      gtctttaaggttcttggtcaaaacgaccaattgatgtggttatctcttggatattggttc    780
TH985       gtctttaaggttcttggtcaaaacgaccaattgatgtggttatctcttggatattggttc    780
1F8CT       gtctttaaggttcttggtcaaaacgaccaattgatgtggttatctcttggatattggttc    780
TH982       gtctttaaggttcttggtcaaaacgaccaattgatgtggttatctcttggatattggttc    780
            ******** ********* *********** ******** * ***** ************

TH1477      tatggtcttggtattaatacacttaatgctcttcaactttattacttcacatttatcctt    840
TH1435      tatggtcttggtattaatacacttaatgctcttcaactttattacttcacatttatcctt    840
LMG18311    TATGGTCTTGGTATTAATACACTTAATGCTCTTCAACTTTATTATTTCACATTTATCCTT    840
TH1436      tatggtcttggtattaatacacttaatgctcttcaactttattatttcacatttatcctt    840
TH985       tatggtcttggtattaatacacttaatgctcttcaactttattatttcacatttatcctt    840
1F8CT       tatggtcttggtattaatacacttaatgctcttcaactttattatttcacatttatcctt    840
TH982       tatggtcttggtattaatacacttaatgctcttcaactttattatttcacatttatcctt    840
            ******************************************** ***************

TH1477      ggtgattcagggaaatactcaatcctttacggattgaatacagttgttggtttggtttca    900
TH1435      ggtgattcagggaaatactcaatcctttacggattgaatacagttgttggtttggtttca    900
LMG18311    GGTGATTCAGGTAAATACTCAATTCTTTACGGATTGAATACAGTTGTTGGTTTGGTTTCA    900
TH1436      ggtgattcaggtaaatactcaattctttacggattgaatacagttattggtttggtttca    900
TH985       ggtgattcaggtaaatattcaattctttacggattgaatacagttgttggtttggtttca    900
1F8CT       ggtgattcaggtaaatactcaattctttacggattgaatacagttgttggtttggtttca    900
TH982       ggtgattcaggtaaatactcaattctttacggattgaatacagttgttggtttggtttca    900
            *********** ***** ***** ********************* **************

TH1477      gtttcactgttccctagtcttgctggtaaattcaaccgtaaacgtttgttctatggatgt    960
TH1435      gtttcactgttccctagtcttgctggtaaattcaaccgtaaacgtttgttctatggatgt    960
LMG18311    GTTTCACTCTTCCCTACCCTAGCTGATAAATTCAACCGTAAACGTTTGTTCTACGGATGT    960
TH1436      gtttcactcttccctaccctagctaataaattcaaccgtaaacgtttgttctacggatgt    960
TH985       gtttcactcttccctaccctagctgataaattcaaccgtaaacgtttgttctacggatgt    960
1F8CT       gtttcactcttccctaccctagctgataaattcaaccgtaaacgtttgttctacggatgt    960
TH982       gtttcactcttccctaccctagctgataaattcaaccgtaaacgtttgttatacggatgt    960
            ******** *******  ** ***  ************************ ** ******
```

```
TH1477      attgcagtaatgctcggtggtatcggaatctttagtattgcaggtacttcacttccaatg      1020
TH1435      attgcagtaatgctcggtggtatcggaatctttagtattgcaggtacttcacttccaatg      1020
LMG18311    ATTGCAGTAATGCTCGGGGGTATCGGAATATTTAGTATTGCAGGTACATCACTTCCAATA      1020
TH1436      attgcagtaatgctcggggtatcggaatctttagtattgcaggtacatcacttccaata      1020
TH985       attgcagtaatgctcggggtatcggaatctttagtattgcaggtacatcacttccaata      1020
1F8CT       attgcagtaatgctcggggtatcggaatctttagtattgcaggtacatcacttccaata      1020
TH982       attgcagtaatgctcggggtatcggaatctttagtattgcaggtacatcacttccaata      1020
            ***************** *********** ***************** ***********

TH1477      atcttgactgcagctgaactcttcttcattccacaacctcttgtgttccttgttatcctt      1080
TH1435      atcttgactgcagctgaactcttcttcattccacaacctcttgtgttccttgttatcctt      1080
LMG18311    ATCTTGACTGCAGCTGAACTCTTCTTCATTCCACAACCTCTTGTGTTCCTTGTTGTCTTT      1080
TH1436      atcttgactgcagctgaactcttcttcattccacaacctcttgtgttccttgttgtcttt      1080
TH985       atcttgactgcagctgaactcttcttcattccacaacctcttgtgttccttgttgtcttt      1080
1F8CT       atcttgactgcagctgaactcttcttcattccacaacctcttgtgttccttgttgtcttt      1080
TH982       atcttgactgcagctgaactcttcttcattccacaacctcttgtgttccttgttgtcttt      1080
            ****************************************************** ** **

TH1477      atgattatctctgactcagtagaatatggtcaatggaaattgggacaccgtgatgaatca      1140
TH1435      atgattatctctgactcagtagaatatggtcaatggaaattgggacaccgtgatgaatca      1140
LMG18311    ATGATTATCTCTGACTCAGTAGAATATGGTCAATGGAAAACGGGACACCGTGATGAATCA      1140
TH1436      atgattatctctgactcagtagaatatggtcaatggaaaacgggacaccgtgatgaatca      1140
TH985       atgattatctctgactcagtagaatatggtcaatggaaaacgggacaccgtgatgaatca      1140
1F8CT       atgattatctctgactcagtagaatatggtcaatggaaaacgggacaccgtgatgaatca      1140
TH982       atgattatctctgactcagtagaatatggtcaatggaaaacgggacaccgtgatgaatca      1140
            ***************************************  *******************

TH1477      cttactttgtcagttcgtccacttgttgataaacttggtggtgcgatgtcaaactggctt      1200
TH1435      cttactttgtcagttcgtccacttgttgataaacttggtggtgcgatgtcaaactggctt      1200
LMG18311    CTTACTTTGTCAGTTCGTCCACTTATTGATAAACTTGGTGGTGCGATGTCAAACTGGCTT      1200
TH1436      cttactttgtcagttcgtccacttattgataaacttggtggtgcgatgtcaaactggctt      1200
TH985       cttactttgtcagttcgtccacttattgataaacttggtggtgcgatgtcaaactggctt      1200
1F8CT       cttactttgtcagttcgtccacttattgataaacttggtggtgcgatgtcaaactggctt      1200
TH982       cttactttgtcagttcgtccacttattgataaacttggtggtgcgatgtcaaactggctt      1200
            ************************ ***********************************

TH1477      gtttctacaattgccgtagctgccggtatgacaacaggtgcctcagcatcaacaattaca      1260
TH1435      gtttctacaattgccgtagctgccggtatgacaacaggtgcctcagcatcaacaattaca      1260
LMG18311    GTTTCTACATTTGCCGTAGCTGCCGGTATGACAACAGGTGCCTCAGCATCAACAATTACA      1260
TH1436      gtttctacatttgccgtagctgccggtatgacaacaggtgcctcagcatcaacaattaca      1260
TH985       gtttctacatttgccgtagctgccggtatgacaacaggtgcctcagcatcaacaattaca      1260
1F8CT       gtttctacatttgccgtagctgccggtatgacaacaggtgcctcagcatcaacaattaca      1260
TH982       gtttctacatttgccgtagctgccggtatgacaacaggtgcctcagcatcaacaattaca      1260
            ********* **************************************************

TH1477      acacatcaacagtctatttttaagcttagcatgtttggtttcccagcagcagcaatgctt      1320
TH1435      acacatcaacagtctatttttaagcttagcatgtttgctttcccagcagcagcaatgctt      1320
LMG18311    ACACATCAACAGTTTATCTTTAAGCTTGGCATGTTTGCTTTCCCAGCAGCAACAATGCTT      1320
TH1436      acacatcaacagtttatctttaagcttggcatgtttgctttcccagcagcaacaatgctt      1320
TH985       acacatcaacagtttatctttaagcttggcatgtttgctttcccagcagcaacaatgctt      1320
1F8CT       acacatcaacagtttatctttaagcttggcatgtttgctttcccagcagcaacaatgctt      1320
TH982       acacatcaacagtttatctttaagcttggcatgtttgctttcccagcagcaacaatgctt      1320
            ************* *** ********* ********* ************* ********

TH1477      atcggtgccttcattattgctcgtaaaatcactttgactgaagcacgtcacgctgaaatt      1380
TH1435      atcggtgccttcattattgctcgtaaaatcactttgactgaagcacgtcacgctaaaatt      1380
LMG18311    ATCGGTGCCTTCATTGTTGCTCGTAAAATCACTTTGACTGAAGCACGTCACGCTAAAATT      1380
TH1436      atcggtgccttcattattgctcgtaaagtcactttgactgaagcacgtcacgctaaaatt      1380
TH985       atcggtgccttcattattgctcgtaaaatcactttgactgaagcacgtcacgctaaaatt      1380
1F8CT       atcggtgccttcattattgctcgtaaaatcactttgactgaagcacgtcacgctaaaatt      1380
TH982       atcggtgccttcattattgctcgtaaaatcactttgactgaagcacgtcacgctaaaatt      1380
            *************** *********** ************************** *****

TH1477      gttgaagaattggaaaatcgctttagcgtagcaacttctgaaaatgaagttaaagctaac      1440
TH1435      gttgaagaattggaacatcgctttagcgtagcaacttctgaaaatgaagttaaagctaac      1440
LMG18311    GTTGAAGAATTGGAACATCGCTTTAGCGTAGCAACTTCTGAAAATGAAGTTAAAGCTAAC      1440
TH1436      gttgaagaattggaacatcgctttagcgtagcaacttctgaaaatgaagttaaagctaac      1440
TH985       gttgaagaattggaacatcgctttagcgtagcaacttctgaaaatgaagttaaagctaac      1440
1F8CT       gttgaagaattggaacatcgctttagcgtagcaacttctgaaaatgaagttaaagctaac      1440
TH982       gttgaagaattggaacatcgctttagcgtagcaacttctgaaaatgaagttaaagctaac      1440
            *************** ********************************************
```

```
TH1477      gtcgtatctcttgtagcccctacaactggtaaactagttgatctttcaagcgttagtgat   1500
TH1435      gtcgtatctcttgtaacccctacaactggttatttggttgatctctcaagtgttaatgat   1500
LMG18311    GTCGTATCTCTTGTAACCCCTACAACTGGTTATTTGGTTGATCTCTCAAGTGTTAATGAT   1500
TH1436      gtcgtatctcttgtaacccctacaactggttatttggttgatctctcaagtgttaatgat   1500
TH985       gtcgtatctcttgtaacccctacaactggttatttggttgatctctcaagtgttaatgat   1500
1F8CT       gtcgtatctcttgtaacccctacaactggttatttggttgatctctcaagtgttaatgat   1500
TH982       gtcgtatctcttgtaacccctacaactggttatttggttgatctctcaagtgttaatgat   1500
            *************** ************** *  * ******** ***** **** ****

TH1477      aaacgctttgcttcaggtagcctaggtaaaggatttgcaatcaaaccttcagatggtaag   1560
TH1435      gaacactttgcttcaggtagcatgggtaaaggtttcgccattaaacctactgatggagct   1560
LMG18311    GAACACTTTGCTTCAGGTAGCATGGGTAAAGGTTTCGCCATTAAACCTACTGATGGAGCT   1560
TH1436      gaacactttgcttcaggtagcatgggtaaaggtttcgccattaaacctactgatggagtt   1560
TH985       gaacactttgcttcaggtagcatgggtaaaggtttcgccattaaacctactgatggagct   1560
1F8CT       gaacactttgcttcaggtagcatgggtaaaggtttcgccattaaacctactgatggagct   1560
TH982       gaacactttgcttcaggtagcatgggtaaaggtttcgccattaaacctactgatggagct   1560
             *** **************** * ******** ** ** ** ****** * *****

TH1477      gtttttgccccaattagtggtacagttcgtcagattctacctactcgtcatgcgattggt   1620
TH1435      gtctttgcaccaattagtggtaccattcgtcaaattcttcctactcgccatgcagttggt   1620
LMG18311    GTCTTTGCACCAATTAGTGGTACCATTCGTCAAATTCTTCCTACTCGCCATGCAGTTGGT   1620
TH1436      gtctttgcaccaattagtggtaccattcgtcaaattcttcctactcgccatgcagttggt   1620
TH985       gtctttgcaccaattagtggtaccattcgtcaaattcttcctactcgccatgcagttggt   1620
1F8CT       gtctttgcaccaattagtggtaccattcgtcaaattcttcctactcgccatgcagttggt   1620
TH982       gtctttgcaccaattagtggtaccattcgtcaaattcttcctactcgccatgcagttggt   1620
            ** ***** **************  ******* ***** ******** *****  *****

TH1477      attgaaagtgatgatggtgtcgttgttctcgtacacgttggtattggaacagttaaactt   1680
TH1435      attgaaagtgaagatggtgtcattgttcttatccacgttggcatcggaacagttaaactt   1680
LMG18311    ATTGAAAGTGAAGATGGTGTCATTGTTCTTATCCACGTTGGCATCGGAACAGTTAAACTT   1680
TH1436      attgaaagtgaagatggtgtcattgttcttatccacgttggcatcggaacagttaaactt   1680
TH985       attgaaagtgaagatggtgtcattgttcttatccacgttggcatcggaacagttaaactt   1680
1F8CT       attgaaagtgaagatggtgtcattgttcttatccacgttggcatcggaacagttaaactt   1680
TH982       attgaaagtgaagatggtgtcattgttcttatccacgttggcatcggaacagttaaactt   1680
            *********** ********* *******  * ******** ** ***************

TH1477      aaaggtgaagggttcattagttacgtaaaacaaggtgatcgtgttgaagttggacaaaaa   1740
TH1435      aatggtgaaggattcattagttacgtagaacaaggtgatcgtgttgaagttggacaaaaa   1740
LMG18311    AATGGTGAAGGATTCATTAGTTACGTAGAACAAGGTGATCATGTTGAAGTTGGACAAAAA   1740
TH1436      aatggtgaaggattcattagttacgtagaacaaggtgatcgtgttgaagttggacaaaaa   1740
TH985       aatggtgaaggattcattagttacgtagaacaaggtgatcgtgttgaagttggacaaaaa   1740
1F8CT       aatggtgaaggattcattagttacgtagaacaaggtgatcgtgttgaagttggacaaaaa   1740
TH982       aatggtgaaggattcattagttacgtagaacaaggtgatcgtgttgaagttggacaaaaa   1740
            ** ******** *************** ************ *******************

TH1477      cttcttgagttctggtcaccaattattgagaaaaatggtcttgatgacacagtacttgtc   1800
TH1435      cttcttgagttctggtcaccaattattgagaaaaatggtcttgatgacacagtacttgtc   1800
LMG18311    CTTCTTGAGTTCTGGTCACCAATTATTGAGAAAAATGGTCTTGATGACACAGTACTTGTC   1800
TH1436      cttcttgagttctggtcaccgattattgagaaaaatggtcttgatgacacagtacttgtc   1800
TH985       cttcttgagttctggtcaccaattattgagaaaaatggtcttgatgacacagtacttgtc   1800
1F8CT       cttcttgagttctggtcaccaattattgagaaaaatggtcttgatgacacagtacttgtc   1800
TH982       cttcttgagttctggtcaccaattattgagaaaaatggtcttgatgacacagtacttgtc   1800
            ******************** ***************************************

TH1477      actgtaactaattcagaaaaattcagtgctttccatcttgaacaaaaagttggagaaaag   1860
TH1435      actgtaactaattcagaaaaattcagtgctttccatcttgaacaaaaagttggagaaaag   1860
LMG18311    ACTGTAACTAATTCAGAAAAATTCAGTGCTTTCCATCTTGAACAAAAAGTTGGAGAAAAG   1860
TH1436      actgtaactaattcagaaaaattcagtgctttccatcttgaacaagaagttggagaaaag   1860
TH985       actgtaactaattcagaaaaattcagtgctttccatcttgaacaaaaagttggagaaaag   1860
1F8CT       actgtaactaattcagaaaaattcagtgctttccatcttgaacaaaaagttggagaaaag   1860
TH982       actgtaactaattcagaaaaattcagtgctttccatcttgaacaaaaagttggagaaaag   1860
            ********************************************* **************

TH1477      gtagaagctttgtctgaagttattaccttcaaaaaaggagaataa   1905
TH1435      gtagaagctttgtctgaagttattaccttcaaaaaaggagaataa   1905
LMG18311    GTAGAAGCTTTGTCTGAAGTTATTACCTTCAAAAAAGGAGAATAA   1905
TH1436      gtagaagctttgtctgaagttattaccttcaaaaaaggagaataa   1905
TH985       gtagaagctttgtctgaagttattaccttcaaaaaaggagaataa   1905
1F8CT       gtagaagctttgtctgaagttattaccttcaaaaaaggagaataa   1905
TH982       gtagaagctttgtctgaagttattaccttcaaaaaaggagaataa   1905
            *********************************************
```

lacZ

```
TH1477          atgaacacgactgaaaaaattcaaacttacttaaacgatccaaagattgttagcgttaat     60
LMG18311        ATGAACATGACTGAAAAAATTCAAACTTATTTAAACGATCCAAAGATTGTTAGCGTTAAT     60
TH985           atgaacatgactgaaaaaattcaaacttatttaaacgatccaaagattgttagcgttaat     60
TH1435          atgaacatgactgaaaaaattcaaacttatttaaacgatccaaagattgttagcgttaat     60
TH982           atgaacatgactgaaaaaattcaaacttatttaaacgatccaaagattgttagcgttaat     60
TH1436          atgaacacgactgaaaaaattcaaacttacttaaacgatccaaagattgttagcgttaat     60
1F8CT           atgaacatgactgaaaaaattcaaacttatttaaacgatccaaagattgttagcgttaat     60
                ******* ********************* ******************************

TH1477          actgttgacgctcactcagatcataagtattttgaatctcttgcagaattttctgagggg     120
LMG18311        ACTGTTGATGCTCACTCAGATCATAAGTATTTTGAATCTCTTGAAGAATTTTCTGAAGGG     120
TH985           actgttgatgctcactcagatcataagtattttgaatctcttgaagaattttctgaaggg     120
TH1435          actgttgatgctcactcagatcataagtattttgaatctcttgaagaattttctgaaggg     120
TH982           actgttgatgctcactcagatcataagtattttgaatctcttgaagaattttctgaaggg     120
TH1436          actgttgacgctcactcagatcataagtattttgaatctcttgcagaattttctgagggg     120
1F8CT           actgttgatgctcactcagatcataagtattttgaatctcttgaagaattttctgaaggg     120
                ******** ********************************** ************ ***

TH1477          gagatgaagttaagacaatctcttaatggaaaatggaaaattcactatgctcagaataca     180
LMG18311        GAGATGAAGTTAAGACAATCTCTTAATGGAAAATGGAAAATTCACTATGCTCAGAATACA     180
TH985           gagatgaagttaagacaatctcttaatggaaaatggaaaattcactatgctcagaataca     180
TH1435          gagatgaagttaagacaatctcttaatggaaaatggaaaattcactatgctcagaataca     180
TH982           gagatgaagttaagacaatctcttaatggaaaatggaaaattcactatgctcagaataca     180
TH1436          gagatgaagttaagacaatctcttaatggaaaatggaaaattcactatgctcagaataca     180
1F8CT           gagatgaagttaagacaatctcttaatggaaaatggaaaattcactatgctcagaataca     180
                ************************************************************

TH1477          aatcaggttttaaaagacttttataaaacagaatttgatgaaactgatttgaatttcatc     240
LMG18311        AATCAGGTTTTAAAAGACTTTTATAAAACAGAATTTGATGAAACTGATTTGAATTTCATC     240
TH985           aatcaggttttaaaagacttttataaaacagaatttgatgaaactgatttgaatttcatc     240
TH1435          aatcaggttttaaaagacttttataaaacagaatttgatgaaactgatttgaatttcatc     240
TH982           aatcaggttttaaaagacttttataaaacagaatttgatgaaactgatttgaatttcatc     240
TH1436          aatcaggttttaaaagacttttataaaacagaatttgatgaaactgatttgaatttcatc     240
1F8CT           aatcaggttttaaaagacttttataaaacagaatttgatgaaactgatttgaatttcatc     240
                ************************************************************

TH1477          aatgtaccaggtcatttagagcttcaaggttttggttctccacaatatgtgaatacccag     300
LMG18311        AATGTACCAGGTCATTTAGAGCTTCAAGGTTTTGGTTCTCCACAATATGTGAATACCCAA     300
TH985           aatgtaccaggtcatttagagcttcaaggttttggttctccacaatatgtgaatacccaa     300
TH1435          aatgtaccaggtcatttagagcttcaaggttttggttctccacaatatgtgaatacccaa     300
TH982           aatgtaccaggtcatttagagcttcaaggttttggttctccacaatatgtgaatacccaa     300
TH1436          aatgtaccaggtcatttagagcttcaaggttttggttctccacaatatgtgaatacccag     300
1F8CT           aatgtaccaggtcatttagagcttcaaggttttggttctccacaatatgtgaatacccaa     300
                ***********************************************************

TH1477          tatccttgggatggtaaagaattccttcgtccacctcaagttcctcaagaatcaaatgct     360
LMG18311        TATCCTTGGGATGGTAAAGAATTCCTTCGTCCACCTCAAGTTCCTCAAGAATCAAATGCT     360
TH985           tatccttgggatggtaaagaattccttcgtccacctcaagttcctcaagaatcaaatgct     360
TH1435          tatccttgggatggtaaagaattccttcgtccacctcaagttcctcaagaatcaaatgct     360
TH982           tatccttgggatggtaaagaattccttcgtccacctcaagttcctcaagaatcaaatgct     360
TH1436          tatccttgggatggtaaagaattccttcgtccacctcaagttcctcaagaatcaaatgct     360
1F8CT           tatccttgggatggtaaagaattccttcgtccacctcaagttcctcaagaatcaaatgct     360
                ************************************************************

TH1477          gttgcatcatacgttaaacattttaccttgaatgatgcattaaaagataaaaaagtattt     420
LMG18311        GTTGCATCATACGTTAAACATTTTACCTTGAATGATGCATTAAAAGATAAAAAAGTATTT     420
TH985           gttgcatcatacgttaaacattttaccttgaatgatgcattaaaagataaaaaagtattt     420
TH1435          gttgcatcatacgttaaacattttaccttgaatgatgcattaaaagataaaaaagtattt     420
TH982           gttgcatcatacgttaaacattttaccttgaatgatgcattaaaagataaaaaagtattt     420
TH1436          gttgcatcatacgttaaacattttaccttgaatgatgcattaaaagataaaaaagtattt     420
1F8CT           gttgcatcatacgttaaacattttaccttgaatgatgcattaaaagataaaaaagtattt     420
                ************************************************************

TH1477          atctcattccaaggggttgctacttccatctttgtatgggtcaatggtaacttcgtagga     480
LMG18311        ATCTCATTCCAAGGGGTTGCTACTTCCATCTTTGTATGGGTCAATGGTAACTTCGTAGGA     480
TH985           atctcattccaaggggttgctacttccatctttgtatgggtcaatggtaacttcgtagga     480
TH1435          atctcattccaaggggttgctacttccatctttgtatgggtcaatggtaacttcgtagga     480
TH982           atctcattccaaggggttgctacttccatctttgtatgggtcaatggtaacttcgtagga     480
TH1436          atctcattccaaggggttgctacttccatctttgtatgggtcaatggtaacttcgtagga     480
1F8CT           atctcattccaaggggttgctacttccatctttgtatgggtcaatggtaacttcgtagga     480
                ************************************************************
```

```
TH1477      tacagtgaagattcatttacacctagtgaatttgaaattagtgattaccttgttgaaggt      540
LMG18311    TACAGTGAAGATTCATTTACACCTAGTGAATTTGAAATTAGTGATTACCTTGTTGAAGGT      540
TH985       tacagtgaagattcatttacacctagtgaatttgaaattagtgattaccttgttgaaggt      540
TH1435      tacagtgaagattcatttacacctagtgaatttgaaattagtgattaccttgttgaaggt      540
TH982       tacagtgaagattcatttacacctagtgaatttgaaattagtgattaccttgttgaaggt      540
TH1436      tacagtgaagattcatttacacctagtgaatttgaaattagtgattaccttgttgaaggt      540
1F8CT       tacagtgaagattcatttacacctagtgaatttgaaattagtgattaccttgttgaaggt      540
            ************************************************************

TH1477      gataacaagttggcggtagctgtttatcgttactctacagcaagctggttggaagaccaa      600
LMG18311    GATAACAAGTTGGCGGTAGCTGTTTATCGTTACTCTACAGCAAGCTGGTTGGAAGACCAA      600
TH985       gataacaagttggcggtagctgtttatcgttactctacagcaagctggttggaagaccaa      600
TH1435      gataacaagttggcggtagctgtttatcgttattctacagcaagctggttggaagaccaa      600
TH982       gataacaagttggcggtagctgtttatcgttactctacagcaagctggttggaagaccaa      600
TH1436      gataacaagttggcggtagctgtttatcgttactctacagcaagctggttggaagaccaa      600
1F8CT       gataacaagttggcggtagctgtttatcgttactctacagcaagctggttggaagaccaa      600
            ******************************** ***************************

TH1477      gacttctggagactttacggtatttttagagatgtttacttgtatgctattccaaaagtt      660
LMG18311    GACTTCTGGAGACTTTACGGTATTTTTAGAGATGTTTACTTGTATGCTATTCCAAAAGTT      660
TH985       gacttctggagactttacggtatttttagagatgtttacttgtatgctattccaaaagtt      660
TH1435      gacttctggagactttacggtatttttagagatgtttacttgtatgctattccaaaagtt      660
TH982       gacttctggagactttacggtatttttagagatgtttacttgtatgctattccaaaagtt      660
TH1436      gacttctggagactttacggtatttttagagatgtttacttgtatgctattccaaaagtt      660
1F8CT       gacttctggagactttacggtatttttagagatgtttacttgtatgctattccaaaagtt      660
            ************************************************************

TH1477      cacgttcaagatctctttgttaagggagattatgattaccaaacaaaaacaggtcaattg      720
LMG18311    CACGTTCAAGATCTCTTTGTTAAGGGAGATTATGATTACCAAACAAAAGCAGGTCAATTG      720
TH985       cacgttcaagatctctttgttaagggagattatgattaccaaacaaaagcaggtcaattg      720
TH1435      cacgttcaagatctctttgttaagggagattatgattaccaaacaaaagcaggtcaattg      720
TH982       cacgttcaagatctctttgttaagggagattatgattaccaaacaaaagcaggtcaattg      720
TH1436      cacgttcaagatctctttgttaagggagattatgattaccaaacaaaagcaggtcaattg      720
1F8CT       cacgttcaagatctctttgttaagggagattatgattaccaaacaaaagcaggtcaattg      720
            ************************************************ ***********

TH1477      gatattgatttgaagactgttggtgattatgaagacaagaagattaaatatgttctttca      780
LMG18311    GATATTGATTTGAAGACTGTTGGTGATTATGAAGACAAGAAGATTAAATATGTTCTTTCA      780
TH985       gatattgatttgaagactgttggtgattatgaagacaagaagattaaatatgttctttca      780
TH1435      gatattgatttgaagactgttggtgattatgaagacaagaagattaaatatgttctttca      780
TH982       gatattgatttgaagactgttggtgattatgaagacaagaagattaaatatgttctttca      780
TH1436      gatattgatttgaagactgttggtgattatgaagacaagaagattaaatatgttctttca      780
1F8CT       gatattgatttgaagactgttggtgattatgaagacaagaagattaaatatgttctttca      780
            ************************************************************

TH1477      gattatgaaggcatcgttacagaaggtgatgcatctgttaatggtgacggtgaactatct      840
LMG18311    GATTATGAAGGCATCGTTACAGAAGGTGATGCATCTGTTAATGGTGACGGTGAACTATCT      840
TH985       gattatgaaggcatcgttacagaaggtgatgcatctgttaatggtgacggtgaactatct      840
TH1435      gattatgaaggcatcgttacagaaggtgatgcatctgttaatggtgacggtgaactatct      840
TH982       gattatgaaggcatcgttacagaaggtgatgcatctgttaatggtgacggtgaactatct      840
TH1436      gattatgaaggcatcgttacagaaggtgatgcatctgttaatggtgacggtgaactatct      840
1F8CT       gattatgaaggcatcgttacagaaggtgatgcatctgttaatggtgacggtgaactatct      840
            ************************************************************

TH1477      gtaagtcttgaaaatcttaaaatcaaaccttggagtgctgaaagtcctaaactttacgat      900
LMG18311    GTAAGTCTTGAAAATCTTAAAATCAAACCTTGGAGTGCTGAAAGTCCTAAACTTTACGAT      900
TH985       gtaagtcttgaaaatcttaaaatcaaaccttggagtgctgaaagtcctaaactttacgat      900
TH1435      gtaagtcttgaaaatcttaaaatcaaaccttggagtgctgaaagtcctaaactttacgat      900
TH982       gtaagtcttgaaaatcttaaaatcaaaccttggagtgctgaaagtcctaaactttacgat      900
TH1436      gtaagtcttgaaaatcttaaaatcaaaccttggagtgctgaaagtcctaaactttacaat      900
1F8CT       gtaagtcttgaaaatcttaaaatcaaaccttggagtgctgaaggtcctaaactttacgat      900
            ****************************************** ************** **

TH1477      ttgatccttcatgttttggatgatgaccaagttgttgaagtcgttccagttaaagttgga      960
LMG18311    TTGATCCTTCATGTTTTGGATGATGACCAAGTTGTTGAAGTCGTTCCAGTTAAAGTTGGA      960
TH985       ttgatccttcatgttttggatgatgaccaagttgttgaagtcgttccagttaaagttgga      960
TH1435      ttgatccttcatgttttggatgatgaccaagttgttgaagtcgttccagttaaagttgga      960
TH982       ttgatccttcatgttttggatgatgaccaagttgttgaagtcgttccagttaaagttgga      960
TH1436      ttgatccttcatgttttggatgatgaccaagttgttgaagtcgttccagttaaagttgga      960
1F8CT       ttgatccttcatgttttggatgatgaccaagttgttgaagtcgttccagttaaagttgga      960
            ************************************************************
```

```
TH1477      tttagacgctttgaaattaaagataaacttatgcttttgaatggtaagagaattatcttt    1020
LMG18311    TTTAGACGCTTTGAAATTAAAGATAAACTTATGCTTTTGAATGGTAAGAGAATTGTCTTT    1020
TH985       ttcagacgctttgaaattaaagataaacttatgcttttgaatggtaagagaattgtcttt    1020
TH1435      tttagacgctttgaaattaaagataaacttatgcttttgaatggtaagagaattgtcttt    1020
TH982       tttagacgctttgaaattaaagataaacttatgcttttgaatggtaagagaattgtcttt    1020
TH1436      tttagacgctttgaaattaaagatagacttatgcttttgaatggtaagagaattgtcttt    1020
1F8CT       tttagacgctttgaaattaaagataaacttatgcttttgaatggtaagagaattgtcttt    1020
            ** ********************** **************************** *****

TH1477      aaaggggttaacagacacgaatttaacgctagaacaggacgttgtatcactgaagaagat    1080
LMG18311    AAAGGGGTTAACAGACACGAATTTAACGCTAGAACAGGACGTTGTATCACTGAAGAAGAT    1080
TH985       aaaggggttaacagacacgaatttaacgctagaacaggacgttgtatcactgaagaagat    1080
TH1435      aaaggggttaacagacacgaatttaacgctagaacaggacgttgtatcactgaagaagat    1080
TH982       aaaggggttaacagacacgaatttaacgctagaacaggacgttgtatcactgaagaagat    1080
TH1436      aaaggggttaacagacacgaatttaatgctagaacaggacgttgtatcactgaagaagat    1080
1F8CT       aaaggggttaacagacacgaatttaacgctagaacaggacgttgtatcactgaagaagat    1080
            ************************** *********************************

TH1477      atgctttgggatatcaaagtgatgaaacaacataacatcaatgctgttcgtacttcacac    1140
LMG18311    ATGCTTTGGGATATCAAAGTGATGAAACAACATAACATCAATGCTGTTCGTACTTCACAC    1140
TH985       atgctttgggatatcaaagtgatgaagcaacataacatcaatgctgttcgtacttcacac    1140
TH1435      atgctttgggatatcaaagtgatgaaacaacataacatcaatgctgttcgtacttcacac    1140
TH982       atgctttgggatatcaaagtgatgaaacaacataacatcaatgctgttcgtacttcacac    1140
TH1436      atgctttgggatatcaaagtgatgaaacaacataacatcaatgctgttcgtacttcacac    1140
1F8CT       atgctttgggatatcaaagtgatgaaacaacataatatcaatgctgttcgtacttcacac    1140
            ************************** ******** ************************

TH1477      tatcctaaccaaacacgttggtatgaattgtgtgatgaatatggactttatgttatcgat    1200
LMG18311    TATCCTAACCATACACGTTGGTATGAATTGTGTGATGAATATGGACTTTATGTTATCGAT    1200
TH985       tatcctaaccaaacacgttggtatgaattgtgtgatgaatatggactttatgttatcgat    1200
TH1435      tatcctaaccaaacacgttggtatgaattgtgtgatgaatatggactttatgttatcgat    1200
TH982       tatcctaaccaaacacgttggtatgaattgtgtgatgaatatggactttatgttatcgat    1200
TH1436      tatcctaaccaaacacgttggtatgaattgtgtgatgaatatggactttatgttatcgat    1200
1F8CT       tatcctaaccaaacacgttggtatgaattgtgtgatgaatatggactttatgttatcgat    1200
            *********** ************************************************

TH1477      gaagccaaccttgaaacacacggtacatggcaaaaacttggtctatgcgaaccttcatgg    1260
LMG18311    GAAGCCAACCTTGAAACACACGGTACATGGCAAAAACTTGGTCTATGCGAACCTTCATGG    1260
TH985       gaagccaaccttgaaacacacggtacatggcaaaaacttggtctatgcgaaccttcatgg    1260
TH1435      gaagccaaccttgaaacacacggtacatggcaaaaacttggtctatgcgaaccttcatgg    1260
TH982       gaagccaaccttgaaacacacggtacatggcaaaaacttggtctatgcgaaccttcatgg    1260
TH1436      gaagccaaccttgaaacacacggtacatggcaaaaacttggtctatgcgaaccttcatgg    1260
1F8CT       gaagccaaccttgaaacacacggtacatggcaaaaacttggtctatgcgaaccttcatgg    1260
            ************************************************************

TH1477      aatatcccagctagtgaaccagaatggttgcctgcttgtttggatcgtgccaataacatg    1320
LMG18311    AATATCCCAGCTAGTGAACCAGAATGGTTGCCTGCTTGTTTGGATCGTGCCAATAACATG    1320
TH985       aatatcccagctagtgaaccagaatggttgcctgcttgtttggatcgtgccaataacatg    1320
TH1435      aatatcccagctagtgaaccagaatggttgcctgcttgtttggatcgtgccaataacatg    1320
TH982       aatatcccagctagtgaaccagaatggttgcctgcttgtttggatcgtgccaataacatg    1320
TH1436      aatatcccagctagtgaaccagaatggttgcctgcttgtttggatcgtgccaataacatg    1320
1F8CT       aatatcccagctagtaaaccagaatggttgcctgcttgtttggatcgtgccaataacatg    1320
            *************** ********************************************

TH1477      ttccaacgcgataagaaccacgctagtgttatcatttggtcttgtggtaatgaatcatat    1380
LMG18311    TTCCAACGCGATAAGAACCACGCTAGTGTTATCATTTGGTCTTGTGGTAATGAATCATAT    1380
TH985       ttccaacgcgataagaaccacgctagtgttatcatttggtcttgtggtaatgaatcatat    1380
TH1435      ttccaacgcgataagaaccacgctagtgttatcatttggtcttgtggtaatgaatcatat    1380
TH982       ttccaacgcgataagaaccacgctagtgttatcatttggtcttgtggtaatgaatcatat    1380
TH1436      ttccaacgcgataagaaccacgctagtgttatcatttggtcttgtggtaatgaatcatat    1380
1F8CT       ttccaacgcgataagaaccacgctagtgttatcatttggtcttgtggtaatgaatcatat    1380
            ************************************************************

TH1477      gctggtaaagatattgctgacatggctgattacttccgtagtgttgacaatactcgtcca    1440
LMG18311    GCTGGTAAAGATATTGCTGACATGGCTGATTACTTCCGTAGTGTTGACAATACTCGTCCA    1440
TH985       gctggtaaagatattgctgacatggctgattacttccgtagtgttgacaatactcgtcca    1440
TH1435      gctggtaaagatattgctgacatggctgattacttccgtagtgttgacaatactcgtcca    1440
TH982       gctggtaaagatattgctgacatggctgattacttccgtagtgttgacaatactcgtcca    1440
TH1436      gctggtaaagatattgctgacatggctgattatttccgtagtgttgacaatactcgtcca    1440
1F8CT       gctggtaaagatattgctgacatggctgattatttccgtagtgttgacaatactcgtcca    1440
            ******************************** ***************************
```

```
TH1477      gttcactatgaaggtgttgcatggtgtcgtgagtttgattacattacagacatcgaaagt   1500
LMG18311    GTTCACTATGAAGGTGTTGCATGGTGTCGTGAGTTTGATTACATTACAGACATCGAAAGT   1500
TH985       gttcactatgaaggtgttgcatggtgtcgtgagtttgattacattacagacatcgaaagt   1500
TH1435      gttcactatgaaggtgttgcatggtgtcgtgagtttgattacattacagacatcgaaagt   1500
TH982       gttcactatgaaggtgttgcatggtgtcgtgagtttgattacattacagacatcgaaagt   1500
TH1436      gttcactatgaaggtgttgcatggtgtcgtgagtttgattacattacagacatcgaaagt   1500
1F8CT       gttcactatgaaggtgttgcatggtgtcgtgagtttgattacattacagacatcgaaagt   1500
            ************************************************************

TH1477      cgtatgtatgcgaaaccagctgatatcgaagaatacctcacaactggtaaactagttgat   1560
LMG18311    CGTATGTATGCGAAACCAGCTGATATCGAAGAATACCTCACAACTGGTAAACTAGTTGAT   1560
TH985       cgtatgtatgcgaaaccagctgatatcgaagaatacctcacaactggtaaactagttgat   1560
TH1435      cgtatgtatgcgaaaccagctgatatcgaagaatacctcacaactggtaaactagttgat   1560
TH982       cgtatgtatgcgaaaccagctgatatcgaagaatacctcacaactggtaaactagttgat   1560
TH1436      cgtatgtatgcgaaaccagctgatatcgaagaatacctcacaactggtaaactagttgat   1560
1F8CT       cgtatgtatgcgaaaccagctgatatcgaagaatacctcacaactggtaaactagttgat   1560
            ************************************************************

TH1477      ctttcaagcgttagtgataaacactttgcttcaggtaacctaactaacaaacctcaaaaa   1620
LMG18311    CTTTCAAGCGTTAGTGATAAACACTTTGCTTCAGGTAACCTAACTAACAAACCTCAAAAA   1620
TH985       ctttcaagcgttagtgataaacactttgcttcaggtaacctaactaacaaacctcaaaaa   1620
TH1435      ctttcaagcgttagtgataaacactttgcttcaggtaacctaactaacaaacctcaaaaa   1620
TH982       ctttcaagcgttagtgataaacactttgcttcaggtaacctaactaacaaacctcaaaaa   1620
TH1436      ctttcaagagttagtgataaacactttgcttcaggtaacctaactaacaaacctcaaaaa   1620
1F8CT       ctttcaagcgttagtgataaacactttgcttcaggtaacctaactaacaaacctcaaaaa   1620
            ******** ***************************************************

TH1477      ccttatatttcatgtgaatacatgcacacaatgggtaactctggtggtggattgcaactc   1680
LMG18311    CCTTATATTTCATGTGAATACATGCACACAATGGGTAACTCTGGTGGTGGATTGCAACTC   1680
TH985       ccttatatttcatgtgaatacatgcacacaatgggtaactctggtggtggattgcaactc   1680
TH1435      ccttatatttcatgtgaatacatgcacacaatgggtaactctggtggtggattgcaactc   1680
TH982       ccttatatttcatgtgaatacatgcacacaatgggtaactctggtggtggattgcaactc   1680
TH1436      ccttatatttcatgtgaatacatgcacacaatgggtaactctggtggtggattggaactc   1680
1F8CT       ccttatatttcatgtgaatacatgcacacaatgggtaactctggtggtggattggaactc   1680
            ****************************************************** *****

TH1477      tacactgacttagagaaatatccagaataccaaggtggatttatttgggacttcattgac   1740
LMG18311    TACACTGACTTAGAGAAATATCCAGAATACCAAGGTGGATTTATTTGGGACTTCATTGAC   1740
TH985       tacactgacttagagaaatatccagaataccaaggtggatttatttgggacttcattgac   1740
TH1435      tacactgacttagagaaatatccagaataccaaggtggatttatttgggacttcattgac   1740
TH982       tacactgacttagagaaatatccagaataccaaggtggatttatttgggacttcattgac   1740
TH1436      tacactgacttagagaaatatccagaataccaaggtggatttatttgggacttcattgac   1740
1F8CT       tacactgacttagagaaatatccagaataccaaggtggatttatttgggacttcattgac   1740
            ************************************************************

TH1477      caagctatttacaaaacacttccaaatggtagcgaattcctatcatatggtggtgactgg   1800
LMG18311    CAAGCTATTTACAAAACACTTCCAAATGGTAGCGAATTCCTATCATATGGTGGTGACTGG   1800
TH985       caagctatttacaaaacacttccaaatggtagcgaattcctatcatatggtggtgactgg   1800
TH1435      caagctatttacaaaacacttccaaatggtagcgaattcctatcatatggtggtgactgg   1800
TH982       caagctatttacaaaacacttccaaatggtagcgaattcctatcatatggtggtgactgg   1800
TH1436      caagctatttacaaaacacttccaaatggtagcgaattcctatcatatggtggtgactgg   1800
1F8CT       caagctatttacaaaacacttccaaatggtagcgaattcctatcatatggtggtgactgg   1800
            ************************************************************

TH1477      catgatagaccttctgactacgaattttgtggaaatggtatcgtctttgcagatcgtacc   1860
LMG18311    CATGATAGACCTTCTGACTACGAATTTTGTGGAAATGGTATCGTCTTTGCAGATCGTACC   1860
TH985       catgatagaccttctgactacgaattttgtggaaatggtatcgtctttgcagatcgtacc   1860
TH1435      catgatagaccttctgactacgaattttgtggaaatggtatcgtctttgcagatcgtacc   1860
TH982       catgatagaccttctgactacgaattttgtggaaatggtatcgtctttgcagatcgtacc   1860
TH1436      catgatagaccttctgactacgaattttgtggaaatggtattgtctttgcagatcgtacc   1860
1F8CT       catgatagaccttctgactacgaattttgtggaaatggtattgtctttgcagatcgtacc   1860
            ***************************************** ******************

TH1477      ctaactccaaaacttcaaacagttaaacatctttactctaatattaagattgctgttgat   1920
LMG18311    CTAACTCCAAAACTTCAAACAGTTAAACATCTTTACTCTAATATTAAGATTGCTGTTGAT   1920
TH985       ctaactccaaaacttcaaacagttaaacatctttactctaatattaagattgctgttgat   1920
TH1435      ctaactccaaaacttcaaacagttaaacatctttactctaatattaagattgctgttgat   1920
TH982       ctaactccaaaacttcaaacagttaaacatctttactctaatattaagattgctgttgat   1920
TH1436      ctaactccaaaacttcaaacagttaaacatctttactctaatattaagattgctgttgat   1920
1F8CT       ctaactccaaaacttcaaacagttaaacatctttactctaatattaagattgctgttgat   1920
            ************************************************************
```

```
TH1477      gaaaaatcagtaactatcaagaatgataatctcttcgaagatctttctgcttatactttc   1980
LMG18311    GAAAAATCAGTAACTATCAAGAATGATAATCTCTTCGAAGATCTTTCTGCTTATACTTTC   1980
TH985       gaaaaatcagtaactatcaagaatgataatctcttcgaagatctttctgcttatactttc   1980
TH1435      gaaaaatcagtaactatcaagaatgataatctcttcgaagatctttctgcttatactttc   1980
TH982       gaaaaatcagtaactatcaagaatgataatctcttcgaagatctttctgcttatactttc   1980
TH1436      gaaaaatcagtaactatcaagaatgataatgtcttcgaagatctttctgcttatactttc   1980
1F8CT       gaaaaatcagtaactatcaagaatgataatgtcttcgaagatctttctgcttatactttc   1980
            ****************************** *****************************

TH1477      ctagctagagtttacgaagatggtagaaaagttagtgaaagtgaatatcactttgatgtt   2040
LMG18311    CTAGCTAGAGTTTACGAAGATGGTAGAAAAGTTAGTGAAAGTGAATATCACTTTGATGTT   2040
TH985       ctagctagagtttacgaagatggtagaaaagttagtgaaagtgaatatcactttgatgtt   2040
TH1435      ctagctagagtttacgaagatggtagaaaagttagtgaaagtgaatatcactttgatgtt   2040
TH982       ctagctagagtttacgaagatggtagaaaagttagtgaaagtgaatatcactttgatgtt   2040
TH1436      ctagctagagtttacgaagatggtagaaaagttagtgaaagtgaatatcactttgatgtt   2040
1F8CT       ctagctagagtttacgaagatggtagaaaagttagtgaaagtgaatatcactttgatgtt   2040
            ************************************************************

TH1477      aaaccaggcgaagaagcaacattcccagttaactttgtagttgaggcttcaaattctgaa   2100
LMG18311    AAACCAGGCGAAGAAGCAACATTCCCAGTTAACTTTGTAGTCGAGGCTTCAAATTCTGAA   2100
TH985       aaaccaggcgaagaagcaacattcccagttaactttgtagtcgaggcttcaaattctgaa   2100
TH1435      aaaccaggcgaagaagcaacattcccagttaactttgtagtcgaggcttcaaattctgaa   2100
TH982       aaaccaggcgaagaagcaacattcccagttaactttgtagtcgaggcttcaaattctgaa   2100
TH1436      aaaccaggcgaagaagcaacattcccagttaactttgtagtcgaggcttcaaattctgaa   2100
1F8CT       aaaccaggcgaagaagcaacattcccagttaactttgtagtcgaggcttcaaattctgaa   2100
            ***************************************** ******************

TH1477      caaatttacgaagttgcttgtgttctgagggaagcaactaaatgggctcctaaaggtcat   2160
LMG18311    CAAATTTACGAAGTTGCTTGTGTTCTGAGGGAAGCAACTGAATGGGCTCCTAAAGGTCAT   2160
TH985       caaatttacgaagttgcttgtgttctgagggaagcaactgaatgggctcctaaaggtcat   2160
TH1435      caaatttacgaagttgcttgtgttctgagggaagcaactgaatgggctcctaaaggtcat   2160
TH982       caaatttacgaagttgcttgtgttctgagggaagcaactgaatgggctcctaaaggtcat   2160
TH1436      caaatttacgaagttgcttgtgttctgagggaagcaactgaatgggctcctaaaggtcat   2160
1F8CT       caaatttacgaagttgcttgtgttctgagggaagcaactgaatgggctcctaaaggtcat   2160
            *************************************** ********************

TH1477      gaaattgttcgtggtcaatatgttgttgaaaagattagcactgaaacaccagttaaagca   2220
LMG18311    GAAATTGTTCGTGGTCAATATGTTGTTGAAAAGATTAGCACTGAAACACCAGTTAAAGCA   2220
TH985       gaaattgttcgtggtcaatatgttgttgaaaagattagcactgaaacaccagttaaagca   2220
TH1435      gaaattgttcgtggtcaatatgttgttgaaaagattagcactgaaacaccagttaaagca   2220
TH982       gaaattgttcgtggtcaatatgttgttgaaaagattagcactgaaacaccagttaaagca   2220
TH1436      gaaattgttcgtggtcaatatgttgttgaaaagattagcactgaaacaccagtcaaagca   2220
1F8CT       gaaattgttcgtggtcaatatgttgttgaaaagattagcactgaaacaccagtcaaagca   2220
            ***************************************************** ******

TH1477      cctttgaatgttgttgaaggggacttcaacatcggtattcaaggacaaaacttctcaatc   2280
LMG18311    CCTTTGAATGTTGTTGAAGGCGACTTCAACATCGGTATTCAAGGACAAAACTTCTCAATC   2280
TH985       cctttgaatgttgttgaaggcgacttcaacatcggtattcaaggacaaaacttctcaatc   2280
TH1435      cctttgaatgttgttgaaggcgacttcaacatcggtattcaaggacaaaacttctcaatc   2280
TH982       cctttgaatgttgttgaaggcgacttcaacatcggtattcaaggacaaaacttctcaatc   2280
TH1436      cctttgaatgttgttgaaggtgacttcaacatcggtattcaaggacaaaacttctcaatc   2280
1F8CT       cctttgaatgttgttgaaggtgacttcaacatcggtattcaaggacaaaacttctcaatc   2280
            ******************** ***************************************

TH1477      ttgctttcacgtgcacaaaatactttagtatctgctaagtataatggtgttgaattcatt   2340
LMG18311    TTGCTTTCACGTGCACAAAATACTTTAGTATCTGCTAAGTATAATGGTGTTGAATTCATT   2340
TH985       ttgctttcacgtgcacaaaatactttagtatctgctaagtataatggtgttgaattcatt   2340
TH1435      ttgctttcacgtgcacaaaatactttagtatctgctaagtataatggtgttgaattcatt   2340
TH982       ttgctttcacgtgcacaaaatactttagtatctgctaagtataatggtgttgaattcatt   2340
TH1436      ttgctttcacgtgcacaaaatactttagtatctgctaagtataatggtgttgaattcatt   2340
1F8CT       ttgctttcacgtgcacaaaatactttagtatctgctaagtataatggtgttgaattcatt   2340
            ************************************************************
```

```
TH1477      gagaaaggtcctaaacttagcttcactcgtgcttacactgacaacgatcgtggtgctgga      2400
LMG18311    GAGAAAGGTCCTAAACTTAGCTTCACTCGTGCTTACACTGACAACGATCGTGGTGCTGGA      2400
TH985       gagaaaggtcctaaacttagcttcactcgtgcttacactgacaacgatcgtggtgctgga      2400
TH1435      gagaaaggtcctaaacttagcttcactcgtgcttacactgacaacgatcgtggtgctgga      2400
TH982       gagaaaggtcctaaacttagcttcactcgtgcttacactgacaacgatcgtggtgctgga      2400
TH1436      gagaaaggtcctaaacttaacttcactcgtgcttacactgacaacgatcgtggtgctgga      2400
1F8CT       gagaaaggtcctaaacttaacttcactcgtgcttacactgacaacgatcgtggtgctgga      2400
            ******************* ****************************************

TH1477      tatccattcgaaatggcaggctggaaggttgctggaaactatagtaaagttacagatact      2460
LMG18311    TATCCATTCGAAATGGCAGGCTGGAAGGTTGCTGGAAACTATAGTAAAGTTACAGATACT      2460
TH985       tatccattcgaaatggcaggctggaaggttgctggaaactatagtaaagttacagatact      2460
TH1435      tatccattcgaaatggcaggctggaaggttgctggaaactatagtaaagttacagatact      2460
TH982       tatccattcgaaatggcaggctggaaggttgctggaaactatagtaaagttacagatact      2460
TH1436      tatccattcgaaatggcaggatggaaggttgctggaaactatagtaaagttacagatact      2460
1F8CT       tatccattcgaaatggcaggatggaaggttgctggaaactatagtaaagttacagatact      2460
            ******************** ***************************************

TH1477      caaattcaaatcgaagacgactctgttaaagtgacttatgttcatgaattgccaggcttg      2520
LMG18311    CAAATTCAAATCGAAGACGACTCTGTTAAAGTGACTTATGTTCATGAATTGCCAGGCTTG      2520
TH985       caaattcaaatcgaagacgactctgttaaagtgacttatgttcatgaattgccaggcttg      2520
TH1435      caaattcaaatcgaagacgactctgttaaagtgacttatgttcatgaattgccaggcttg      2520
TH982       caaattcaaatcgaagacgactctgttaaagtgacttatgttcatgaattgccaggcttg      2520
TH1436      caaattcaaatcgaagacgactctgttaaagtgacttatgttcatgaattgccaggcttg      2520
1F8CT       caaattcaaatcgaagacgactctgttaaagtgacttatgttcatgaattgccaggcttg      2520
            ************************************************************

TH1477      tctgatgtcgaagttaaggtaacttatcaagttgactacaagggtcgaatctttgttact      2580
LMG18311    TCTGATGTCGAAGTTAAGGTAACTTATCAAGTTGATTACAAGGGTCGAATCTTTGTTACT      2580
TH985       tctgatgtcgaagttaaggtaacttatcaagttgattacaagggtcgaatctttgttact      2580
TH1435      tctgatgtcgaagttaaggtaacttatcaagttgattacaagggtcgaatctttgttact      2580
TH982       tctgatgtcgaagttaaggtaacttatcaagttgattacaagggtcgaatctttgttact      2580
TH1436      tctgatgtcgaagttaaggtaacttatcaagttgactacaagggtcgaatctttgttact      2580
1F8CT       tctgatgtcgaagttaaggtaacttatcaagttgactacaagggtcgaatctttgttact      2580
            *********************************** ************************

TH1477      gcaaactatgatggtaaagcaggtttgccaaacttccctgaatttggtctagaatttgct      2640
LMG18311    GCAAACTATGATGGTAAAGCAGGTTTGCCAAACTTCCCTGAATTTGGTTTAGAATTTGCT      2640
TH985       gcaaactatgatggtaaagcaggtttgccaaacttccctgaatttggtctagaatttgct      2640
TH1435      gcaaactatgatggtaaagcaggtttgccaaacttccctgaatttggtctagaatttgct      2640
TH982       gcaaactatgatggtaaagcaggtttgccaaacttccctgaatttggtctagaatttgct      2640
TH1436      gcaaactatgatggtaaagcaggtttgccaaacttccctgaatttggtctagaatttgct      2640
1F8CT       gcaaactatgatggtaaagcaggtttgccaaacttccctgaatttggtctagaatttgct      2640
            ************************************************ ***********

TH1477      atcggttcacaatttacaaaccttagctattatggatacggtgcagaagaaagctaccgt      2700
LMG18311    ATCGGTTCACAATTTACAAACCTTAGCTATTATGGATACGGTGCAGAAGAAAGCTACCGT      2700
TH985       atcggttcacaatttacaaaccttagctattatggatacggtgcagaagaaagctaccgt      2700
TH1435      atcggttcacaatttacaaaccttagctattatggatacggtgcagaagaaagctaccgt      2700
TH982       atcggttcacaatttacaaaccttagctattatggatacggtgcagaagaaagctaccgt      2700
TH1436      atcggttcacaatttacaaaccttagctattatggatacggtgcagaagaaagctaccgt      2700
1F8CT       atcggttcacaatttacaaaccttagctattatggatacggtgcagaagaaagctactgt      2700
            ********************************************************* **

TH1477      gataaacttcctggtgcctatcttggtcgatatgaaacatctgttgaaaagacatttgct      2760
LMG18311    GATAAACTTCCTGGTGCCTATCTTGGTCGATATGAAACATCTGTTGAAAAGACATTTGCT      2760
TH985       gataaacttcctggtgcctatcttggtcgatatgaaacatctgttgaaaagacatttgct      2760
TH1435      gataaacttcctggtgcctatcttggtcgatatgaaacatctgttgaaaagacatttgct      2760
TH982       gataaacttcctggtgcctatcttggtcgatatgaaacatctgttgaaaagacatttgct      2760
TH1436      gataaacttcctggtgcctatcttggtcgatatgaaacatctgttgaaaagacatttgct      2760
1F8CT       gataaacttcctggtgcctatcttggtcgatatgaaacatctgttgaaaagacatttgct      2760
            ************************************************************

TH1477      ccatatctaatgccacaagaatctggtaatcactatggtactcgtgaattcacagtatct      2820
LMG18311    CCATATCTAATGCCACAAGAATCTGGTAATCACTATGGTACTCGTGAATTCACAGTATCT      2820
TH985       ccatatctaatgccacaagaatctggtaatcactatggtactcgtgaattcacagtatct      2820
TH1435      ccatatctaatgccacaagaatctggtaatcactatggtactcgtgaattcacagtatct      2820
TH982       ccatatctaatgccacaagaatctggtaatcactatggtactcgtgaattcacagtatct      2820
TH1436      ccatatctaatgccacaagaatctggtaatcactatggtactcgtgaattcacagtatct      2820
1F8CT       ccatatctaatgccacaagaatctggtaatcactatggtactcgtgaattcacagtatct      2820
            ************************************************************
```

```
TH1477        gatgataaccataatggtcttaaattcaccgcacttaataaagcattcgaattcagtgct      2880
LMG18311      GATGATAACCATAATGGTCTTAAATTCACCGCACTTAATAAAGCATTCGAATTCAGTGCT      2880
TH985         gatgataaccataatggtcttaaattcaccgcacttaataaagcattcgaattcagtgct      2880
TH1435        gatgataaccataatggtcttaaattcaccgcacttaataaagcattcgaattcagtgct      2880
TH982         gatgataaccataatggtcttaaattcaccgcacttaataaagcattcgaattcagtgct      2880
TH1436        gatgataaccataatggtcttaaattcaccgcacttaataaagcattcgaattcagtgct      2880
1F8CT         gatgataaccataatggtcttaaattcaccgcacttaataaagcattcgaattcagtgct      2880
              ************************************************************

TH1477        ttgcgtaacagtactgaacaaattgaaaatgctcgtcaccaatatgagttgcaagaatct      2940
LMG18311      TTGCGTAACACTACTGAACAAATTGAAAATGCTCGTCAACAATATGAGTTGCAAGAATCT      2940
TH985         ttgcgtaacagtactgaacaaattgaaaatgctcgtcaccaatatgagttgcaagaatct      2940
TH1435        ttgcgtaacagtactgaacaaattgaaaatgctcgtcaccaatatgagttgcaagaatct      2940
TH982         ttgcgtaacagtactgaacaaattgaaaatgctcgtcaccaatatgagttgcaagaatct      2940
TH1436        ttgcgtaacagtactgaacaaattgaaaatgctcgtcaccaatatgagttgcaagaatct      2940
1F8CT         ttgcgtaacagtactgaacaaattgaaaatgctcgtcaccaatatgagttgcaagaatct      2940
              ********** *************************** *********************

TH1477        gatgctacatggattaaagttcttgctgctcaaatgggtgtaggtggtgacgactcatgg      3000
LMG18311      GATGCTACATGGATTAAAGTGCTTGCTGCTCAAATGGGTGTAGGTGGTGACGACACATGG      3000
TH985         gatgctacatggattaaagttcttgctgctcaaatgggtgtcggtggtgacgacacatgg      3000
TH1435        gatgctacatggattaaagttcttgctgctcaaatgggtgtaggtggtgacgacacatgg      3000
TH982         gatgctacatggattaaagttcttgctgctcaaatgggtgtcggtggtgacgacacatgg      3000
TH1436        gatgctacatggattaaagttcttgctgctcaaatgggtgtaggtggtgacgactcatgg      3000
1F8CT         gatgctacatggattaaagttcttgctgctcaaatgggtgtaggtggtgacgactcatgg      3000
              ******************** ******************** ************ *****

TH1477        ggtgctccagttcatgacgaattcttgcttagctcagcagatagctatcaattaagcttc      3060
LMG18311      GGTGCTCCAGTTCATGACGAATTCTTGCTTAGCTCAGCAGATAGCTATCAATTAAGCTTC      3060
TH985         ggtgctccagttcatgacgaattcttgcttagctcagcagatagctatcaattaagcttc      3060
TH1435        ggtgctccagttcatgacgaattcttgcttagctcagcagatagctatcaattaagcttc      3060
TH982         ggtgctccagttcatgacgaattcttgcttagctcagcagatagctatcaattaagcttc      3060
TH1436        ggtgctccagttcatgacgaattcttgcttagctcagcagatagctatcaattaagcttc      3060
1F8CT         ggtgctccagttcatgacgaattcttgcttagctcagcagatagctatcaattaagcttc      3060
              ************************************************************

TH1477        atgattgaaccactaaattag     3081
LMG18311      ATGATTGAACCACTAAATTAG     3081
TH985         atgattgaaccactaaattag     3081
TH1435        atgattgaaccactaaattag     3081
TH982         atgattgaaccactaaattag     3081
TH1436        atgattgaaccactaaattag     3081
1F8CT         atgattgaaccactaaattag     3081
              *********************
```
